# Semi-Synthetic H_2_S Releasing Compounds with Antioxidant and Vasorelaxant Properties

**DOI:** 10.1021/acsmedchemlett.5c00624

**Published:** 2025-12-08

**Authors:** Valentina Citi, Antonino N. Fallica, Loredana Salerno, Nicola F. Virzì, Valeria Ciaffaglione, Sebastiano Intagliata, Sara Veneziano, Giada Benedetti, Jacopo Spezzini, Alma Martelli, Vincenzo Calderone, Valeria Pittalà

**Affiliations:** † Department of Pharmacy, 9310 University of Pisa, Via Bonanno 6, 56126 Pisa, Italy; ‡ Interdepartmental Research Center, “Biology and Pathology of Ageing”, 9310 University of Pisa, Via Risorgimento 36, 56126 Pisa, Italy; § Department of Drug and Health Sciences, 9298 University of Catania, Viale Andrea Doria 6, 95125 Catania, Italy

**Keywords:** Hydrogen sulfide, oxidative stress, Nrf2, heme oxygenase-1, 4-octylitaconate, hypertension

## Abstract

Hypertension represents a severe cardiovascular pathology linked to the increase in reactive oxygen species that impair blood vessel function. Herein, we report on the synthesis of hybrid compounds designed to release H_2_S and incorporate natural or semisynthetic scaffolds capable of activating the Nrf2 pathway. The molecular hybrids enable a multitarget approach concurrently inducing vasorelaxation upon H_2_S release and mitigating oxidative stress through Nrf2-dependent antioxidant responses via the upregulation of cytoprotective proteins, including HO-1. The itaconate derivative **8b** displayed an optimal H_2_S release in both amperometric and cellular assays. In human aortic smooth muscle cells, compound **8b** counteracted ROS production and cytotoxicity in H_2_O_2_-injured cells and led to the activation of potassium channels with consequent cell hyperpolarization and vasorelaxation, which was also observed in isolated rat aortic rings. Overall, our findings indicate that simultaneous Nrf2 activation and H_2_S release hold significant potential as a new therapeutic strategy for the treatment of hypertension.

## Graphical abstract

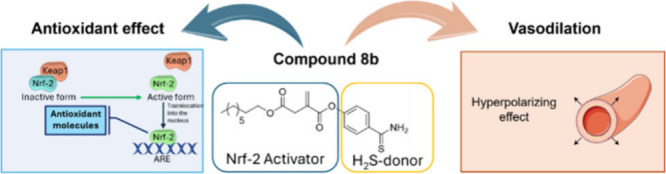

Hypertension is a common medical condition in which the blood force against the walls of the arteries is consistently too high. Often called the “silent killer”, it may not have noticeable symptoms but can cause serious long-term damage to the heart, kidneys, brain, and other organs.^1^ Hypertension is often associated with oxidative stress (OS).^2–4^ The relationship between hypertension and OS is bidirectional: OS contributes to the development of hypertension and sustained high blood pressure in turn exacerbates oxidative damage.^5,6^ The excessive production of ROS leads to endothelial dysfunction, impairing the production of nitric oxide (NO), a key molecule supporting blood vessels relaxation and dilatation, allowing for smooth blood flow.^7–9^ Blood vessels become stiffer and narrower, increasing resistance to blood flow, which can raise blood pressure.^10–12^ Developing new therapeutic strategies that target both OS and high blood pressure represents a promising strategy to reduce the risk of cardiovascular diseases. Current antihypertensive treatments effectively reduce blood pressure by modulating vascular tone and cardiac output; however, they are often characterized by side effects and do not directly address the underlying causes that contribute to long-term cardiovascular damage.

Hydrogen sulfide (H_2_S) is a gasotransmitter which contributes to cardiovascular health by directly promoting vasodilation, as it activates different classes of potassium channels, for example ATP-sensitive potassium channels (K_ATP_ channels), voltage-gated potassium channels (Kv7 channels) and Ca^2+^-activated potassium channels (KCa channels).^13–15^ Also, H_2_S inhibits phosphodiesterase type 5 (PDE5)^16,17^ leading to an increase in cGMP levels, which further contributes to vasodilation and blood pressure regulation.^18–20^ Natural and synthetic molecules able to donate this gasotransmitter have been described to contribute to lowering blood pressure, improving endothelial function, and protecting the cardiovascular system.^21–25^ H_2_S also interacts with the ROS-mediated OS response network and plays an important role in the maintenance of stable redox equilibrium neutralizing ROS, thereby preventing oxidative damage in endothelial cells.^26–28^ However, nuclear factor erythroid derived 2 (Nrf2) is probably the ‘master regulator’ of the antioxidant response that should be targeted to fully counteract OS. In an activated state, Nrf2 stimulates the expression of hundreds of genes, most of them encoding for antioxidant/detoxifying enzymes, with heme-oxygenase 1 (HO-1) being one of the most important.^29–31^ Thus, the pharmacologic induction of Nrf2 activity is regarded as a good strategy for counteracting the OS.

Drug combination therapy is commonly recognized as an effective method for enhancing the clinical efficacy of medications through either additive or synergistic effects. However, the simultaneous use of multiple drugs can lead to challenges such as reduced patient adherence and an increased risk of drug–drug interactions. As a result, there has been growing interest in the development of multitarget ligands acting simultaneously on various biological targets, offering a potential solution to address the limitations associated with coadministering multiple drugs.

Accordingly, the idea underlying this first exploratory series is to merge in one single molecule a double activity, derived from an H_2_S releasing moiety and Nrf2 activators (Figure 1). To this extent we linked together an arylthioamide H_2_S donor moiety such as 4-hydroxybenzothioamide (BTA, compound **6**)^32^ or 4-carbamothioylbenzoic acid (compound **7**), and natural or synthetic compounds known to activate the Nrf2/HO-1 pathway (compounds **1**–**5**).^32–39^

**Figure 1:**
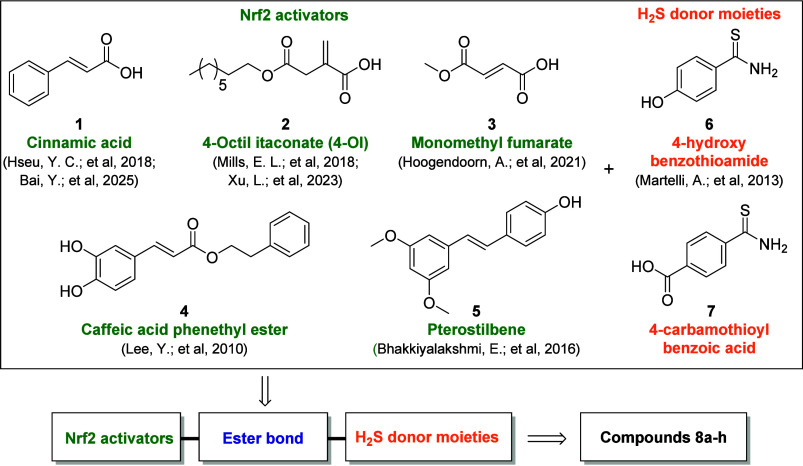
Chemical structures of selected Nrf2 activators, **1**–**5**, and H_2_S donor moieties, **6** and **7**, used for the design of new H_2_S releasing compounds, **8a**–**8h**.

Activators of the Nrf2/HO-1 pathway were selected based on their potency and on the presence of a suitable anchoring point for the H_2_S releasing moiety, such as an esterifiable carboxylic acid moiety or a free hydroxyl group prone to esterification. Selected activators share the common presence of an electrophilic Michael acceptor group, such as the α,β-unsaturated carbonyl system, which enables a Michael addition to cysteine residues of the Kelch-like ECH-associated protein 1 (Keap1), which is the regulator of Nrf2 nuclear translocation.^40,41^ HO-1 expression is cascaded by Nrf2 translocation into the nucleus. Selected Nrf2 activator compounds included cinnamic acid (compound **1**), 4-octyl itaconate (4-OI, compound **2**), fumaric acid, monomethyl fumarate (compound **3**), caffeic acid phenethyl ester (CAPE, compound **4**), pterostilbene (compound **5**), and semi-synthetic derivatives previously reported by our research group.^33,40,42–46^ Compound **6** was previously reported as a long-lasting H_2_S releasing agent at low concentrations. Its H_2_S production mainly relies on the presence of intracellular thiols; however, the mechanism underlying this process is unknown. Compound **6** also displayed a negligible H_2_S release in aqueous environment, pointing out that hydrolytic processes are not primarily responsible for its H_2_S generation.^32^ Overall, compound **6** is an efficient H_2_S releasing agent with a higher hydrolytic stability and sustained temporal H_2_S release at controlled concentrations, avoiding toxic effects related to rapid hydrolysis and lower therapeutic efficacy.

Synthesis of cinnamic acid– and 4-OI–H_2_S releasing hybrids **8a**, **8b** was achieved by direct condensation of 4-hydroxybenzothioamide with the carboxylic acid function of the proper Nrf2/HO-1 inducer moiety (Scheme 1). Compound **2** (4-OI) was synthesized as reported in the literature.^38^

**Scheme 1:**
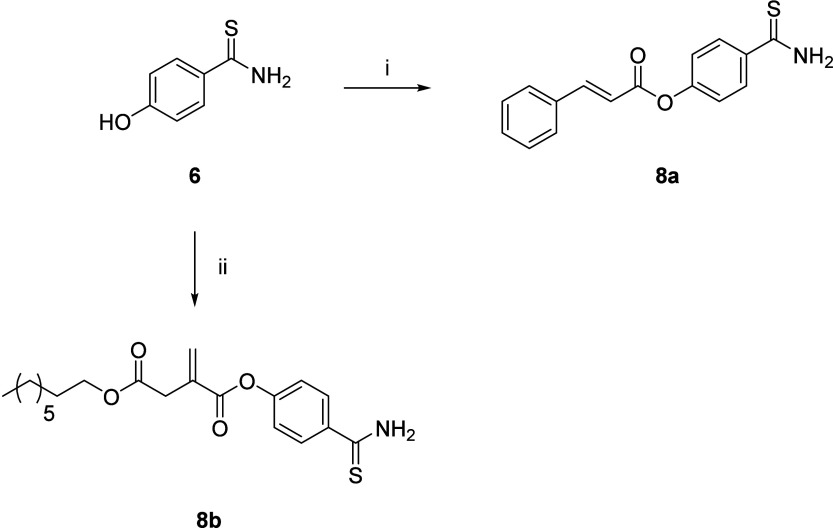
Reagents and Conditions: (i) Cinnamic Acid (Compound**1**), EDC·HCl, DMAP, Dichloromethane, 0 °C, Then Room Temperature Overnight; (ii) 4-OI (Compound**2**), DCC, DMAP, Dichloromethane, 0 °C, then Room Temperature, under Ar, Overnight

Compound **8c** was synthesized by direct coupling between monomethyl fumarate **3** and 4-hydroxybenzothioamide **6** in the presence of EDC hydrochloride, HOBt, and DMAP (Scheme 2). Synthesis of fumarate derivatives **8d**–**8f** was achieved in three steps (Scheme 2). Amidation of monomethyl fumarate with an appropriate amine (aniline, benzylamine, or 4-Cl-benzylamine) afforded intermediates **9a**–**9c**. Then, the ester group of **9a**–**c** was hydrolyzed to obtain **10a**–**10c**. Finally, the re-esterification of the carboxylic acid with 4-hydroxybenzothioamide afforded the final products **8d**–**8f**.

**Scheme 2:**
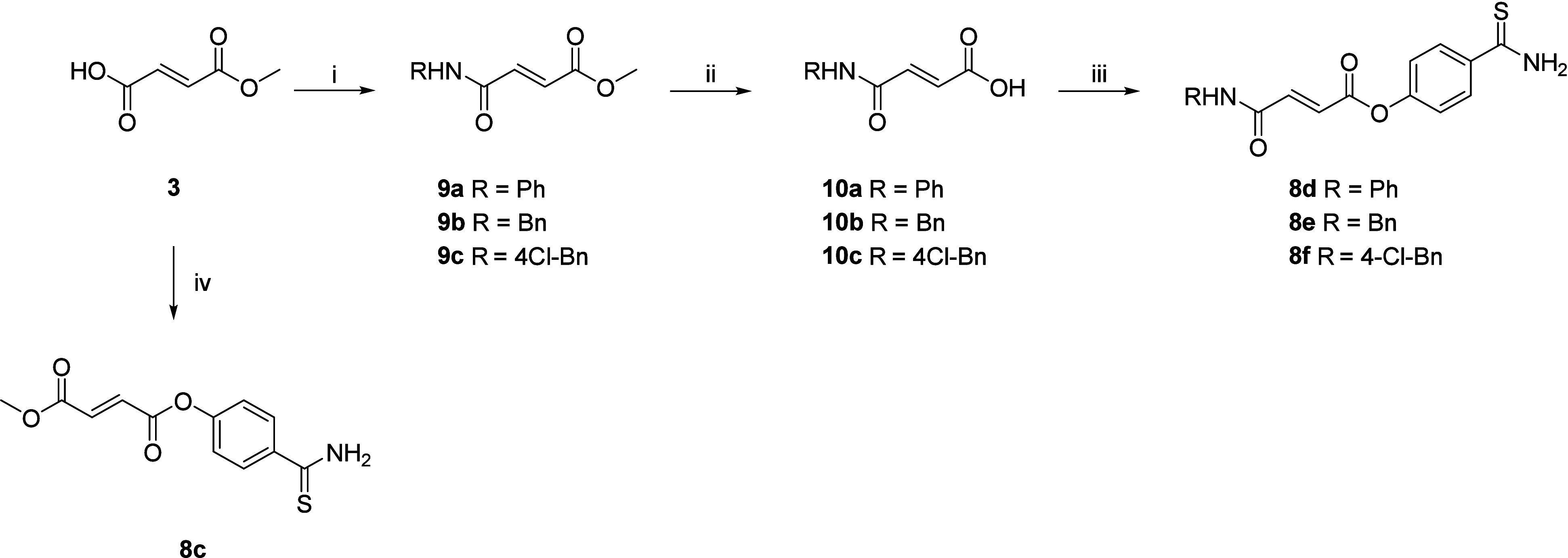
Reagents and Conditions: (i) Appropriate Amine, EDC·HCl, HOBt, Dry DMF, 0 °C, then Room Temperature, under Ar, 16 h; (ii) LiOH·H_2_O, H_2_O/CH_3_OH, Room Temperature, 2 h; (iii) 4-Hydroxybenzothioamide (Compound**6**), EDC·HCl, HOBt, Dry DMF, 0 °C, then Room Temperature, under Ar, 16 h; (iv) 4-Hydroxybenzothioamide (Compound**6**), EDC·HCl, HOBt, DMAP, Dry DMF, 0 °C, then Room Temperature, under Ar, 48 h

Synthesis of CAPE– and pterostilbene–H_2_S releasing hybrids is shown in Scheme 3. Thionation of 4-cyanobenzoic acid with P_2_S_5_ in refluxing ethanol, afforded thioamide **7** that underwent esterification reaction with CAPE (compound **4**) phenolic groups or pterostilbene and EDC hydrochloride as carboxyl activating agent (compound **5**), giving compounds **8g** and **8h**, respectively.

**Scheme 3:**
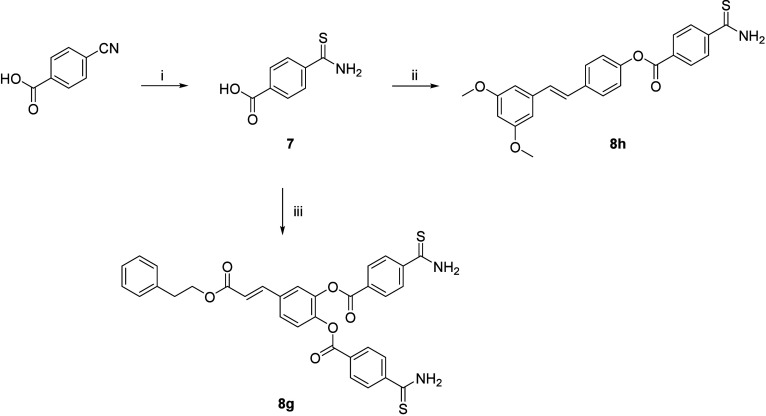
Reagents and Conditions: (i) P_2_S_5_, EtOH, Room Temperature, 1 h, then Reflux, 5 h; (ii) Pterostilbene, EDC·HCl, DMAP, Dry Dichloromethane, 0 °C, then Room Temperature, 16 h, under Ar; (iii) CAPE, EDC·HCl, HOBt, Dry DMF, 0 °C, then Room Temperature, 5 h, under Ar

To investigate the overall drug-likeness of our compounds, ADMET molecular studies were conducted using SwissADME (http://swissadme.ch); results are reported in the Supporting Information (Table S1).^47^ Apart from compound **8g**, compounds are predicted to have moderate (compounds **8b** and **8h**) to good water solubility and high human gastrointestinal absorption. The calculated log *P* values, except for **8g**, showed, for all the compounds, a good hydro/lipophilic balance in a range favorable for absorption across cell membranes but still water-soluble for absorption. All compounds are not predicted to be substrates of the P-glycoprotein nor able to cross the BBB. Most novel H_2_S-donors potentially are inhibitors of cytochromes CYP1A2, CYP2C19, and CYP2C9. Interestingly, only compounds **8b**, **8f**, and **8h** are predicted as CYP3A4 inhibitors, while none of them are foreseen as CYP2D6 inhibitors. Apart from **8g**, compounds have no violation to the Lipinski Rule of 5 and also have no violation to other rules (Ghose, Egan, Veber, and Muegge), suggesting a good drug-likeness profile. Overall, all compounds, excluding **8g**, possess a good druglike profile.

We later evaluated the pharmacological profile of the novel synthesized molecules. An amperometric method, performed in the absence of biological substrates, was chosen as it provides a precise measurement of the H_2_S-releasing process.^48^ The assay was performed in either the absence or presence of l-cysteine (4 mM), used to mimic the endogenous presence of free thiols. All tested compounds at 100 μM exhibited appreciable H_2_S production both in the absence and in the presence of l-cysteine, except for BTA which showed a thiol-dependent H_2_S release. Most compounds released about 1 μM H_2_S, except for **8a**, which generated approximately 4 μM H_2_S (Figure 2). This difference could be attributable to the diverse structural and electronic properties of the Nrf2 activator moieties esterified with the H_2_S releasing agent, which would indirectly alter the electrophilicity of the carbonyl carbon of the thioamide functional group. Moreover, bulkier or more lipophilic moieties esterified with compounds **6** or **7** would limit the release of H_2_S through hydrolytic mechanisms in the aqueous buffer used in the amperometric method, because of steric effects. Considering compound **8a**, the slight electron-withdrawing effect of the conjugated cinnamic acid moiety coupled with the lack of significant steric hindrance and proper hydrophilic balance rationalize the easier nucleophilic attack to the thioamide functional group, with a consequent higher H_2_S production in aqueous buffer compared to the other compounds of the series. The H_2_S-releasing profiles of the compounds reflect ideal H_2_S donors which should provide a sustained, gradual release of H_2_S at physiological levels to ensure prolonged therapeutic effects. A slow H_2_S releasing kinetic is, indeed, more favorable for therapeutic purposes because it allows to avoid eventual adverse effects due to a massive and fast release of H_2_S.^42,49^ Recognized slow-release H_2_S donors like polysulfides derived from garlic (*Allium sativum* L.) and isothiocyanates derived from *Brassicaceae* family exemplify this desirable profile, making them H_2_S-releasing compounds endowed with a plethora of beneficial effects.^50–52^

**Figure 2:**
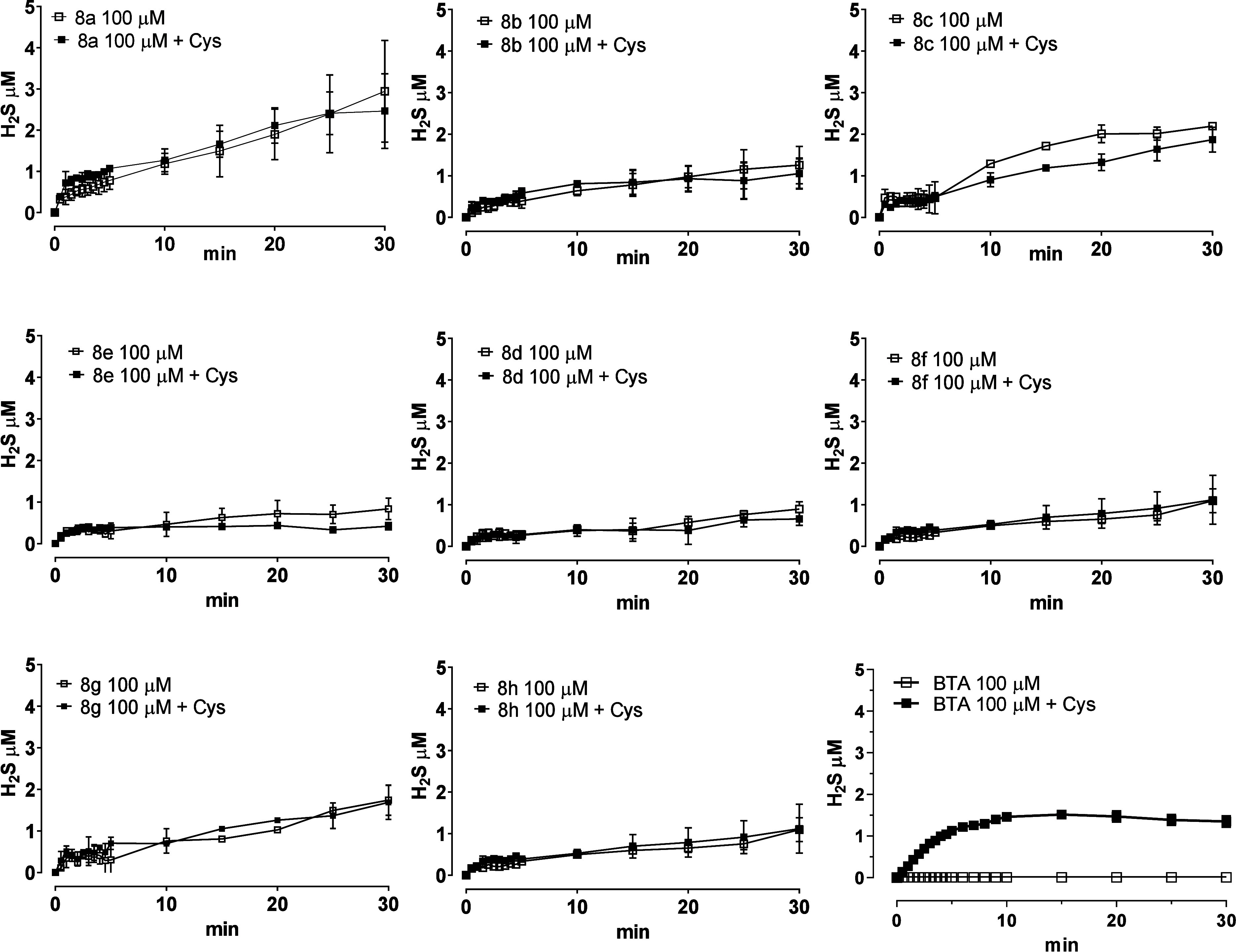
Hydrogen sulfide release with an amperometric approach. The graph shows the H_2_S-release kinetics of the tested compounds. The curves represent the slow increase in H_2_S formation with respect to time. The data are expressed as mean ± SD (*n* = 6).

Human aortic smooth muscle cells (HASMCs) were employed for the detection of intracellular H_2_S release, using the fluorometric Washington State Probe 1 (WSP-1), which specifically and irreversibly interacts with H_2_S.^53,54^ The experiment was conducted without adding exogenous thiols, ensuring that the observed effects reflected the ability of the compounds to intracellularly generate H_2_S. Vehicle (DMSO 1%) promoted a slight increase in fluorescence index (FI), likely due to the endogenous production of H_2_S. Incubation with diallyl disulfide (DADS) 100 μM, used as a reference molecule, resulted in a significant increase in FI, indicating substantial H_2_S generation. The thiobenzamide moiety, as expected, significantly released H_2_S (Figure 3). Among the hybrid molecules, **8a**–**8c** released H_2_S more efficiently than **8d**–**8h**, which showed a negligible increase in intracellular H_2_S donation (Figure 3).

**Figure 3:**
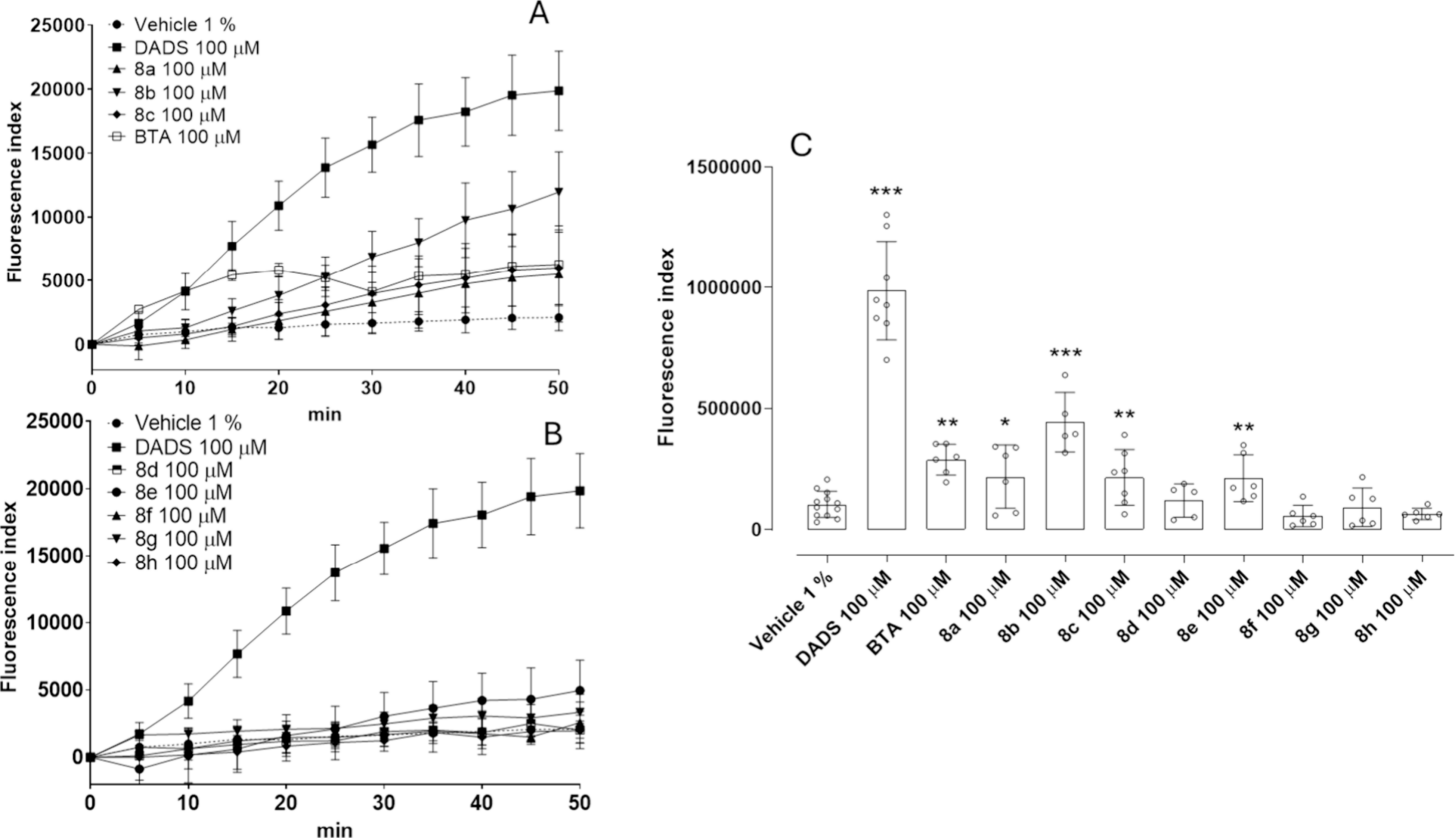
Intracellular H_2_S donation. (A) FI over time in the presence of vehicle 1% (DMSO 1%), DADS, **8a**–**8c** and BTA 100 μM. (B) FI over time in the presence of vehicle 1% (DMSO 1%), DADS, **8d**–**8h** 100 μM. (C) Area under the curve (AUC) of the total amount of H_2_S released in HASMCs over 50 min. Data are shown as mean ± SD (*n* = 3 independent experiments, each performed in triplicate). Statistical significance was assessed by one-way ANOVA followed by Bonferroni post test. Asterisks (*) indicates the significant difference vs vehicle: (*) *p* < 0.05; (**) *p* < 0.01; (***) *p* < 0.001.

The difference in intracellular donation, despite having the same moiety, could be influenced by the physicochemical properties of the molecules, which affect how they cross the cell membrane. Smaller molecules generally diffuse more readily across membranes, while hydrophilic ones may require transporters, as for **8c**. More lipophilic molecules, i.e., compounds with higher log *P* values, tend to passively diffuse through the lipid bilayer more easily, as we can speculate for compounds **8a** and **8b**. The consensus log *P* values estimated by SwissADME supported our experimental observation, suggesting that compounds **8a** and **8b** possess an optimal hydrophilic/lipophilic balance (log *P* equal to 3.19 and 4.24, respectively; see Table S1) that allows sustained intracellular H_2_S release. In addition, the presence of active exporter and importer of 4-OI have been reported,^55^ and, accordingly, we can speculate that the higher ability of compound **8b** to generate intracellular H_2_S could also depend on this mechanism of cell transport.^56^ On the other hand, larger molecules may not permeate cells unless actively transported, as is probably the case for compounds **8g** and **8h**. The higher log *P* values obtained for CAPE derivative **8g** and pterostilbene derivative **8h** (5.37 and 4.60, respectively, Table S1) suggest a reduced intracellular uptake due to higher lipophilicity and a consequent lower H_2_S intracellular release. Hence, given its ability to generate intracellular H_2_S, compound **8b** was selected for further pharmacological investigations using HASMCs.

This derivative of 4-OI, was evaluated to assess the benefits of incorporating a H_2_S-donating moiety in comparison to 4-OI and BTA alone. This modification aims to enhance the protective effects of 4-OI by combining the Nrf2-activating properties of the itaconate derivative with the vasoprotective and vasorelaxant effects of H_2_S. 4-OI already activates Nrf2, leading to the expression of antioxidant genes such as HO-1, NQO1, and GCLC, which counteract oxidative damage.^57^ H_2_S donation from **8b** may further enhance antioxidant defenses by directly scavenging ROS. Exposure to 200 μM H_2_O_2_ provoked a marked reduction in cell viability and a concomitant massive increase in intracellular ROS production (Figures 4A and 4B). Pretreatment with **8b** at 1 μM elicited only a modest and statistically insignificant improvement in viability. However, increasing the concentration to 3 μM resulted in a statistically significant recovery of cell viability. In parallel, both concentrations of **8b** significantly reduced ROS accumulation, suggesting that H_2_S release plays a direct role in mitigating oxidative damage, likely through ROS scavenging. In contrast, treatment with the compounds 4-OI and the H_2_S-releasing moiety BTA, each tested at 1 and 3 μM, produced only a slight, insignificant increase in cell viability, and neither compound was effective in reducing intracellular ROS levels. These findings suggest that, while both agents possess intrinsic biological activity, they cannot protect in short-term incubation. Importantly, BTA alone failed to reduce ROS or improve viability. Similarly, the inability of 4-OI alone to suppress ROS suggests that it is not able to rapidly activate Nrf2. **8b** emerges as the most effective in restoring cell viability and reducing ROS level, exerting significant cell protection, compared with 4-OI and BTA. The significant differences observed in the protective effects of **8b**, 4-OI, and BTA on cell viability and ROS production can likely be attributed to the combination of a H_2_S-donating moiety BTA with 4-OI, in **8b**. A critical feature of **8b** is its lipophilicity conferred by the octyl chain, which facilitates the rapid diffusion across the cell membrane. This property likely underlies the intracellular accumulation of the compound and the acute onset of its protective effects.

**Figure 4:**
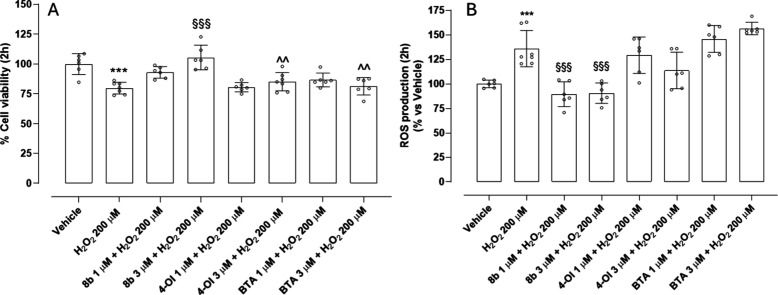
Protective effect of **8b**, 4-OI, and BTA against H_2_O_2_-induced oxidative stress in HASMCs after 2 h of treatment: (A) the vehicle-treated group is set as 100% of cell viability and (B) the vehicle-treated group is set as 100% of ROS production (data are presented as mean ± SD (*n* > 6)). Statistical significance was assessed by one-way ANOVA followed by Bonferroni post test. (*) indicates the statistical significance vs vehicle: (***) *p* < 0.001); (§) indicates the statistical significance vs H_2_O_2_ ((§§) *p* < 0.01); (∧) indicates the statistical significance vs **8b** 3 μM + H_2_O_2_.

To explore the role of Nrf2 in modulating acute oxidative stress responses, cell viability and ROS production were measured following a 2-h exposure to H_2_O_2_ 200 μM, with or without pretreatment with the Nrf2 inhibitor ML385 10 μM. Interestingly, ML385 did not affect viability, and the coadministration with H_2_O_2_ did not significantly exacerbate H_2_O_2_-induced cytotoxicity. However, pretreatment with ML385 significantly enhanced ROS accumulation when incubated with vehicle and a further increase was recorded when incubated with H_2_O_2_, supporting the importance of Nrf2 in controlling oxidative stress, even in the early phases of exposure (see Figures 5A and 5B). This indicates that, despite the enhanced ROS accumulation upon Nrf2 inhibition, the extent and duration of oxidative stress were not sufficient to induce further cytotoxic effects. These findings underscore that ROS elevation is an early and sensitive indicator of oxidative imbalance, while a measurable impact on cell viability likely requires a longer oxidative exposure. Importantly, the protective effect of **8b** was abolished by cotreatment with ML385, indicating that its ROS-lowering activity is largely dependent on Nrf2 activation. Generally, preincubation with ML385 significantly increased the level of ROS production in each treatment, highlighting the central role of Nrf2 in controlling ROS production even after short-term exposure to H_2_O_2_.

**Figure 5:**
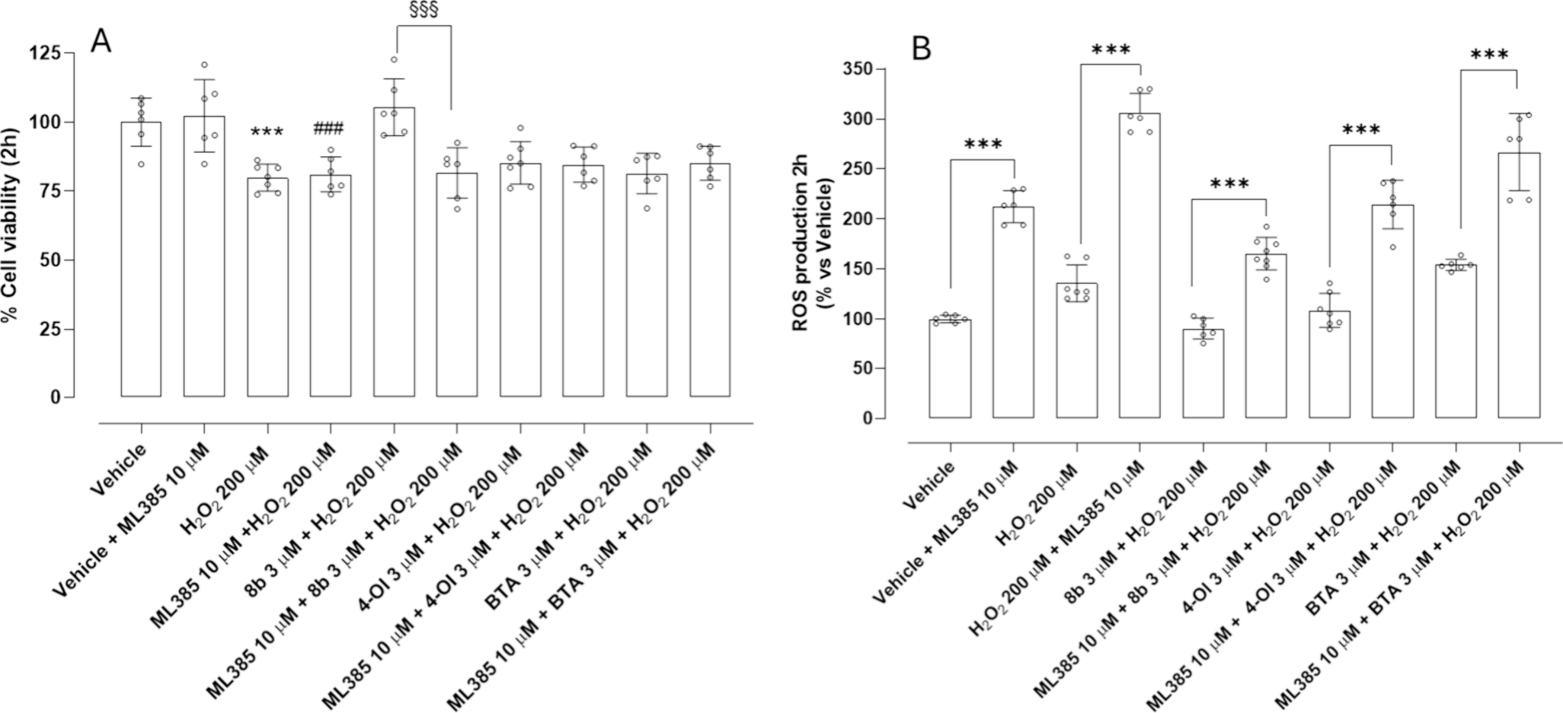
Role of Nrf2 in mediating the protective effect of **8b**, 4-OI and BTA against H_2_O_2_-induced oxidative stress in HASMCs after 2 h of treatment: (A) the vehicle-treated group is set as 100% of cell viability and (B) the vehicle-treated group is set as 100% of ROS production. Data are presented as mean ± SD (*n* > 6). Statistical significance was assessed by one-way ANOVA followed by Bonferroni post test. (*) indicates the statistical significance vs vehicle ((***) *p* < 0.001); (#) indicates the statistical significance vs vehicle + ML385 ((###) *p* < 0.001); (§) indicates the statistical significance vs **8b** 3 μM + H_2_O_2_ ((§§§) *p* < 0.001).

The protective effects of **8b**, 4-OI, and BTA were further assessed following 24 h incubation to evaluate their efficacy under prolonged oxidative stress. The antioxidant and cytoprotective properties observed at early time points were largely preserved after extended exposure, particularly for **8b**, which exhibited significant protection at both 1 and 3 μM. The significant reduction in intracellular ROS levels and preservation of cell viability suggest that **8b** mediates both acute and prolonged protective responses, likely through a combination of H_2_S-dependent redox modulation and Nrf2 activation. Similarly, 4-OI significantly reduced ROS at both concentrations and improved cell viability at 3 μM, reflecting a protecting effect upon incubation for a longer period (see Figures 6A and 6B). BTA, which had shown minimal efficacy in the short-term assays, exerted a significant cytoprotective effect at 3 μM after 24 h, though it remained ineffective in reducing ROS at either concentration. The delayed onset of protection observed with BTA may reflect the time-dependent nature of intracellular H_2_S release and downstream signaling, which becomes evident only under conditions of prolonged exposure.

**Figure 6:**
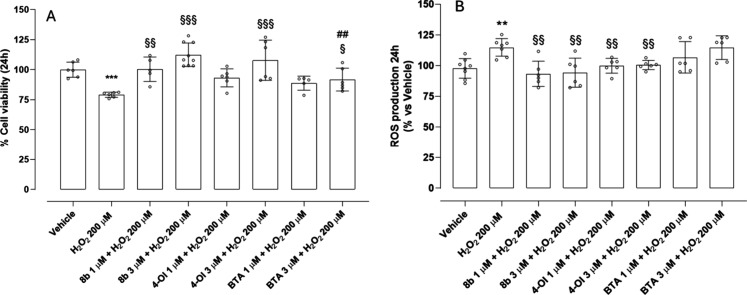
Protective effect of **8b**, 4-OI, and BTA against H_2_O_2_-induced oxidative stress in HASMCs after 24 h of treatment: (A) the vehicle-treated group is set as 100% of cell viability and (B) the vehicle-treated group is set as 100% of ROS production. Data are presented as mean ± SD (*n* > 6). Statistical significance was assessed by one-way ANOVA, followed by Bonferroni post test. (*) indicates the statistical significance vs vehicle ((***) *p* < 0.001); (§) indicates the statistical significance vs H_2_O_2_ ((§§) *p* < 0.01; (§§§) *p* < 0.001); (#) indicates the statistical significance vs **8b** 3 μM + H_2_O_2_ ((##) *p* < 0.01).

To further elucidate the role of the Nrf2 pathway in mediating the antioxidant and cytoprotective effects of **8b**, 4-OI, and BTA, cell viability and intracellular ROS levels were assessed following 24-h exposure to oxidative stress (200 μM H_2_O_2_), in the presence or absence of the Nrf2 inhibitor ML385. As shown in the viability data (Figure 7A), pretreatment with ML385 completely abolished the protective effect of 4-OI 3 μM, strongly supporting that its cytoprotective effect is mainly mediated via Nrf2 activation. In contrast, compound **8b** maintained a significant protective effect, even in the presence of ML385. Although a partial reduction in efficacy was observed—suggesting that its activity is partially Nrf2-dependent—the persistence of protection highlights the contribution of additional, Nrf2-independent mechanisms. This duality may be due to the bifunctional design of **8b**: while the 4-OI scaffold confers Nrf2-activating capacity, the incorporation of a thiobenzamide–H_2_S-releasing moiety adds a second mechanism of cell protection. H_2_S is known to directly scavenge ROS, preserve mitochondrial integrity, and activate prosurvival pathways, including those involving Akt and Nrf2-independent antioxidant enzymes. The effect of BTA 3 μM in cell protection was not affected by Nrf2 inhibition, indicating that its activity is independent of this pathway. However, BTA did not significantly reduce intracellular ROS levels after 24 h. The ROS quantification data (Figure 7B) further reinforced these conclusions. The effect of 4-OI was entirely reversed by the use of ML385. In contrast, **8b** preserved a slight reduction in ROS even with Nrf2 inhibition, suggesting a direct chemical scavenging mechanism via H_2_S release.

**Figure 7:**
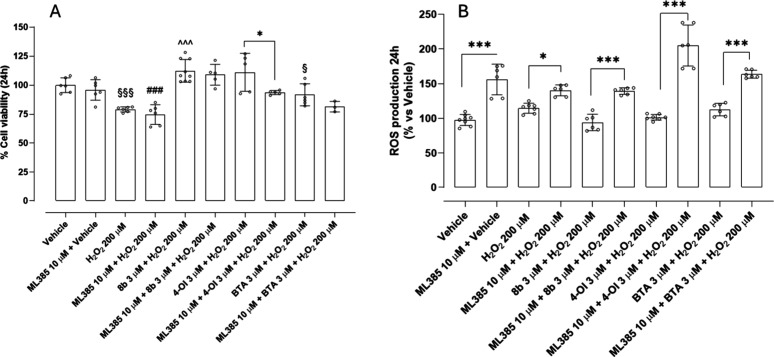
Role of Nrf2 in mediating the protective effect of **8b**, 4-OI, and BTA against H_2_O_2_-induced oxidative stress in HASMCs after 24 h of treatment: (A) the vehicle-treated group is set as 100% of cell viability and (B) the vehicle-treated group is set as 100% of ROS production. Data are presented as mean ± SD (*n* > 6). Statistical significance was assessed by one-way ANOVA followed by Bonferroni post test. (§) indicates the statistical significance vs vehicle ((§§§) *p* < 0.001); (#) indicates the statistical significance vs vehicle + ML385 ((###) *p* < 0.001); (∧) indicates the statistical significance vs H_2_O_2_ ((§) *p* < 0.05; (§§§) *p* < 0.001); (*) indicates the statistical significance as reported over the bars ((*) *p* < 0.05; (**) *p* < 0.01; (***) *p* < 0.001).

The vasorelaxant activity of H_2_S in HASMCs is mediated by multiple mechanisms, including the ability to activate various classes of potassium (K^+^) channels. Activation of these channels leads to membrane hyperpolarization, a process that decreases cell excitability and induces vasodilation. Several studies have demonstrated that H_2_S can directly modulate BKCa, K_ATP_, and Kv7 channels, contributing to smooth muscle relaxation.^13–15^ In hypertension, excessive and uncontrolled contraction of the vascular smooth muscle is a key pathophysiological feature. Given the potential therapeutic relevance of membrane hyperpolarization, the effects of **8b**, 4-OI, and BTA on the membrane potential of cultured HASMCs were evaluated (Figure 8). NS1619, a well-established BKCa potassium channel activator, was used as a reference hyperpolarizing agent.^58^ Compound **8b** exhibited a potent and concentration-dependent hyperpolarizing effect: **8b** (30 μM) induced a hyperpolarization comparable to the reference drug NS1619. **8b** (100 μM) further increased the hyperpolarizing effect significantly surpassing NS1619. Notably, at 300 μM, **8b** induced an even greater hyperpolarization. The observed hyperpolarization suggests that **8b** may act through the activation of potassium channels, leading to a reduction in the cellular excitability and promoting vasodilation. In contrast, 4-OI did not promote membrane hyperpolarization, even at the highest tested concentration of 300 μM. BTA promoted a significant hyperpolarizing effect only when incubated at 300 μM. The observed superiority of **8b** compared with 4-OI and BTA in promoting membrane hyperpolarization may rely on its hybrid structure, in which the thiobenzamide moiety is conjugated to 4-OI. The choice of using HASMCs was based on the known mechanism of action of H_2_S-donors, which exert vasorelaxant effects primarily by activating potassium channels expressed on vascular smooth muscle cells. HASMCs, therefore, provide a mechanistically relevant in vitro model to study the direct cellular effects of H_2_S-releasing compounds.

**Figure 8:**
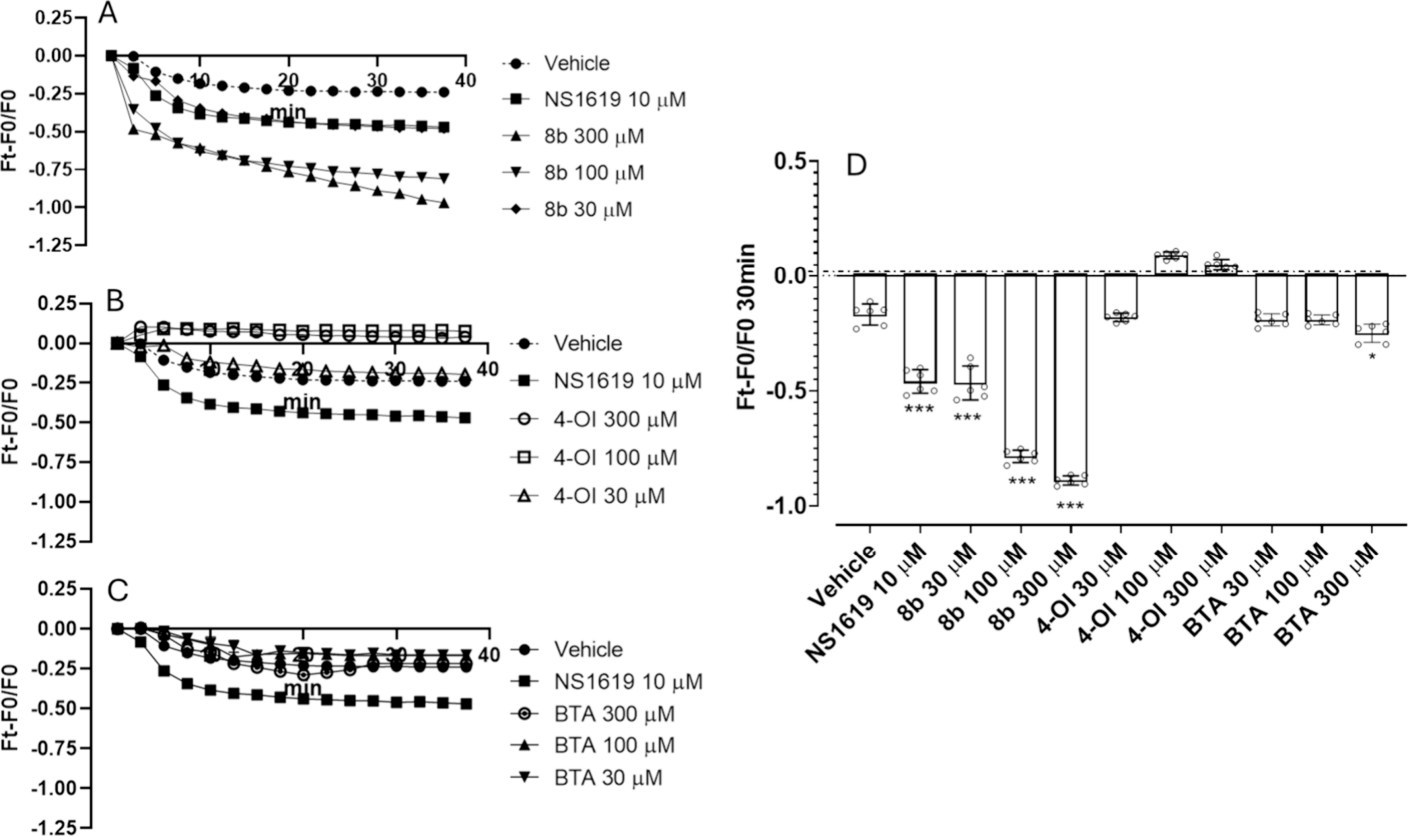
Hyperpolarizing effect of compound **8b**, 4-OI and BTA on HASMCs: (A) hyperpolarization curves over time of vehicle (DMSO 1%), NS1619 10 μM, **8b** 30 μM, 100 μM and 300 μM, (B) 4-OI 30 μM, 100 μM and 300 μM, and (C) BTA 30 μM, 100 μM and 300 μM. (D) Hyperpolarization recorded after 30 min. Data are shown as mean ± SD, (*n* = 3 independent experiments, each performed in triplicate). Statistical significance was assessed by one-way ANOVA followed by Bonferroni post test. (*): statistical significance vs vehicle ((***) *p* < 0.001).

The vasorelaxant effects of **8b**, 4-OI, and BTA were evaluated in isolated rat aortic rings in the absence of endothelium (A) (Figure 9A). When the endothelium was removed, at lower concentrations (ranging between 10^–9^ and 10^–7^ M), **8b**, 4-OI, and BTA exhibited minimal vasorelaxation (data not shown). However, at higher concentrations (10^–5^ and 10^–4^ M), **8b**, 4-OI, and BTA promoted significant vasorelaxation compared with vehicle. Furthermore, **8b** evoked a more pronounced vasorelaxant effect, in comparison with 4-OI and BTA. This is likely due to the additional contribution of H_2_S-mediated mechanisms. While both compounds may share a common pharmacophore responsible for their base vasodilatory activity, the enhanced effect of **8b** suggests that its H_2_S-releasing moiety provides an additive or synergistic contribution to vascular relaxation. Indeed, H_2_S activates K_ATP_ and Kv7 channels in vascular smooth muscle cells, leading to hyperpolarization and reducing smooth muscle contraction and promoting vessel relaxation.^13–15^ To date, this is the first time that the vasorelaxant properties of 4-OI are described. Due to the superiority in vasodilatory effect, **8b** was also evaluated for its ability to inhibit the noradrenaline-induced vasoconstriction in denuded rat aortic rings (Figure 9B). As expected, the vehicle group exhibits the strongest vasoconstrictive response, following a sigmoidal dose–response curve with a steep increase in constriction as the noradrenaline concentration rises. In contrast, **8b** significantly attenuated noradrenaline-induced vasoconstriction, as shown by the lower response curve compared to the vehicle.

**Figure 9:**
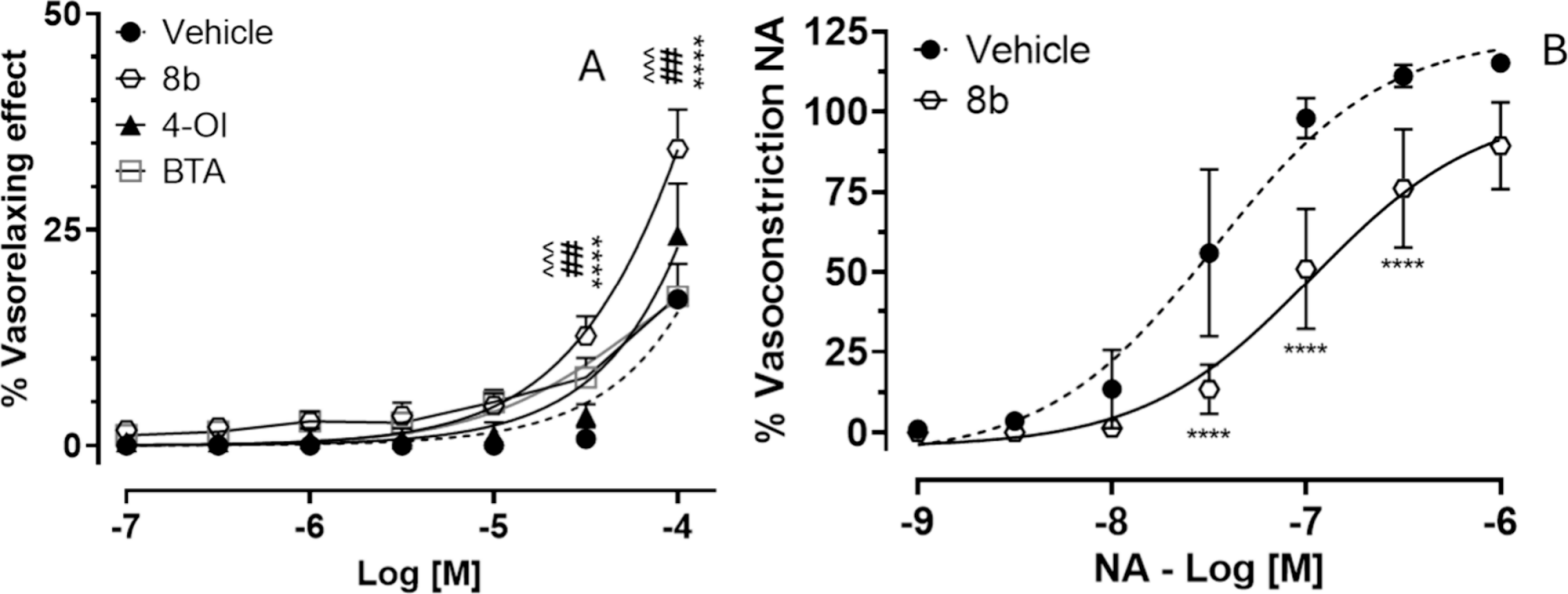
(A) Dose–response curve showing the vasorelaxant effect (%) induced by **8b**, 4-OI and BTA compared to the vehicle in endothelium-denuded isolated rat aortic rings. (B) The inhibitory effect of **8b** in noradrenaline-induced vasoconstriction. (*) indicates significance vs vehicle ((****) *p* < 0.0001). Symbols (#) indicates significance vs 4-OI ((##) *p* < 0.01); (∧) indicates significance vs BTA. Data are expressed as mean ± SD (*n* = 6).

The findings of this study underscore the pivotal role of H_2_S donation in enhancing the cytoprotective properties of **8b**, reinforcing the concept that incorporating H_2_S-releasing moieties into drug design represents a valuable strategy for developing novel antioxidant and cytoprotective agents. The ability of **8b** to release H_2_S in a slow and controlled manner offers a significant therapeutic advantage, particularly in conditions where oxidative stress plays a central role such as hypertension. Moreover, the observed hyperpolarization suggests a mechanism of action involving the activation of potassium channels, which leads to a reduction in cellular excitability and promotes vasodilation. This effect is particularly relevant in the context of vascular health, in regulating vascular tone and reducing blood pressure. The potential of **8b** to modulate these pathways highlights its broader pharmacological relevance, not only as an antioxidant but also as a regulator of vascular function.

## Supplementary Material



## Notes

Experimental procedures, ^1^H NMR and ^13^C NMR spectra of final compounds **8a**–**8h**; in silico ADMET assessment of final compounds **8a**–**8h** (PDF)

## ○

For Antonino N. Fallica: Institute for Bioengineering of Catalonia (IBEC), The Barcelona Institute of Science and Technology, 08028, Barcelona, Spain; and Barcelona Institute for Global Health (ISGlobal), Hospital Clínic–Universitat de Barcelona, 08036 Barcelona, Spain

## ⊥

For Valeria Ciaffaglione: Institute of Crystallography, National Council of Research (CNR), P. Gaifami 18, 95126 Catania, Italy

## ABBREVIATIONS

4-OI: 4-octyl itaconate
ADMET: absorption, distribution, metabolism, excretion, and toxicity
AUC: area under the curve
CAPE: caffeic acid phenethyl ester
DADS: diallyl disulfide
DCC: N,N′-Dicyclohexylcarbodiimide
DCM: dichloromethane
DHE: dihydroethidium
DMAP: 4-dimethylaminopyridine
DMF: dimethylformamide
EDC·HCl: 1-Ethyl-3-(3-(dimethylamino)­propyl)­carbodiimide hydrochloride
EtOAc: ethyl acetate
EtOH: ethanol
FI: fluorescence index
GCLC: Glutamate–cysteine ligase catalytic subunit
HASMCs: human aortic smooth muscle cells
HO-1: heme oxygenase-1
HOBt: hydroxybenzotriazole, K_ATP_, ATP-sensitive potassium channels
KCa: Ca^2+^-activated potassium channels
Keap1: Kelch-like ECH-associated protein 1
Kv7: voltage-gated potassium channels
NQO1: NAD­(P)­H:quinone oxidoreductase 1
Nrf2: Nuclear factor erythroid 2-related factor 2
OS: oxidative stress
PDE5: phosphodiesterase type 5
ROS: reactive oxygen species
TLC: thin-layer chromatography
WSP-1: Washington State Probe 1
