# Supplementary material for: Semi-Synthetic H2S Releasing Compounds with Antioxidant and Vasorelaxant Properties
Source: ACS Med Chem Lett. 2025 Dec 8;17(1):199–210. doi: 10.1021/acsmedchemlett.5c00624 (PMC12794063; doi:10.1021/acsmedchemlett.5c00624)
Supplement: Supplementary file 1 [file ml5c00624_si_001.pdf]

SUPPORTING INFORMATION

Semi-synthetic H_2S releasing compounds with antioxidant and vasorelaxant properties

Valentina Citi[1,2‡], Antonino N. Fallica[3†‡], Loredana Salerno[3], Nicola F. Virzì[3], Valeria Ciaffaglione[3§], Sebastiano Intagliata[3], Sara Veneziano[1], Giada Benedetti[1], Jacopo Spezzini[1], Alma Martelli[1,2], Vincenzo Calderone[1,2], Valeria Pittalà[3*]

[1] *Department of Pharmacy, University of Pisa, Via Bonanno 6, 56126, Pisa, Italy*

[2] *Interdepartmental Research Center "Biology and Pathology of Ageing", University of Pisa, Via Risorgimento 36, Pisa, Italy*

[3] *Department of Drug and Health Sciences, University of Catania, Viale Andrea Doria 6 95125, Catania, Italy*

Corresponding author:

*Valeria Pittalà

Email: valeria.pittala@unict.it

‡ These authors contributed equally to this work and should be considered co-first authors.

Table of contents

Experimental section	S2
^{1}H NMR and ^{13}C NMR spectra of final compounds **8a–h**	S14–
In silico ADMET assessment for compounds **8a–h**	S22

Experimental section

1. Chemistry

CAPE (compound **4**) was synthesized as previously reported.[1] All other reagents and solvents were purchased from commercial vendors and were used without any further modification. Reactions were monitored on TLC aluminum sheets coated with silica gel (60 F254, Merck, Kenilworth, NJ, USA) and visualized by UV (λ = 254 and 366 nm) and iodine chamber. Purification was carried out by flash chromatography on glass columns using Merck silica gel (60, 0.040–0.063 mm, 230–400 mesh) as stationary phase. Melting points were determined in an IA9200 Electrothermal apparatus equipped with a digital thermometer in capillary glass tubes and are uncorrected. Infrared spectra were recorded on a Perkin Elmer 281 FTIR spectrometer using KBr disks. ^{1}H and ^{13}C NMR spectra were recorded using Varian Unity Inova 200 and 500 MHz instruments. Tetramethylsilane (TMS) was used as the internal standard. Chemical shifts are reported in ppm values, coupling constants (*J*) are given in Hz. Signal multiplicities are characterized as s (singlet), d (doublet), t (triplet), q (quartet), m (multiplet), br (broad). Elemental analyses (C, H, N) were carried out on a Carlo Erba Elemental Analyzer Mod. 1108; results were within ±0.4% of the theoretical values.

1.1. Synthesis of 4-carbamothioylbenzoic acid (7)

A flamed round-bottom flask was filled with 6 mL of EtOH. Argon was bubbled into the solvent for 30 minutes, then the temperature was lowered to 0 °C with an ice bath and P_2S_5 (4.08 mmol, 2 eq) was added. The ice bath was removed and the mixture was stirred at room temperature for 1 hour. Subsequently, 4-cyanobenzoic acid (2.04 mmol, 1 eq) was added in one portion and the solution refluxed for 5 hours. After this time, a yellow suspension was obtained. The mixture was poured in ice-cold water and the yellow precipitate was filtered under vacuum. The precipitate was dissolved in EtOAc and the organic phase was washed with $NaHCO_3$. The organic phase was discarded, the aqueous phase was acidified with HCl 1M and extracted three times with EtOAc. The organic phase was dried with Na_2SO_4, filtered and rotavaped. The crude was adsorbed on silica gel and purified by flash chromatography eluting with gradient mixture of CH_2Cl_2 and EtOH. Yellow solid (59%): mp 248–249 °C; IR (KBr) cm^{-1} 3275, 3152, 1684, 1630, 1573, 1506, 1423, 1279, 1107, 1016, 950, 905, 866, 811, 781, 698; ^{1}H NMR (200 MHz, DMSO–d_6) δ 10.01 (s, 1H, thioamide), 9.64 (s, 1H, thioamide), 7.97–7.87 (m, 4H,

aromatic); Anal. Calcd. for $C_8H_7NO_2S$: C, 53.03; H, 3.89; N, 7.73. Found: C, 52.96; H, 3.90; N, 7.70.

*1.2. Synthesis of 4-carbamothioylphenyl cinnamate (**8a**)*

Cinnamic acid (compound **1**, 1.96 mmol, 2 eq) and 4-hydroxybenzothioamide (compound **6**, 0.98 mmol, 1 eq) were dissolved in dichloromethane (10 mL). EDC hydrochloride (1.96 mmol, 2 eq) and DMAP (1.96 mmol, 2 eq) were added at 0 °C and the reaction mixture was left stirring overnight at room temperature. Then, the reaction crude was diluted with EtOAc and washed three times with $NaHCO_3$ and brine. The organic phase was dried over anhydrous Na_2SO_4, filtered, and evaporated under vacuum. The obtained crude material was purified by column chromatography using cyclohexane/EtOAc (7:3) mixture as eluent. Yellow solid (43%): ^{1}H NMR (500 MHz, DMSO–d_6) δ 9.90 (s, 1H, thioamide), 9.54 (s, 1H, thioamide), 7.97 (d, J = 8.5 MHz, 2H, aromatic), 7.90 (d, J = 16.0 Hz, 1H, C*H*=CHCOO), 7.83–7.81 (m, 2H, aromatic), 7.48–7.45 (m, 3H, aromatic), 7.27 (d, J = 8.5 Hz, 2H, aromatic), 6.90 (d, J = 16.0 Hz, 1H, CH=C*H*COO); ^{13}C NMR (125 MHz, DMSO–d_6) δ 199.04, 164.66, 152.66, 146.83, 137.05, 133.79, 130.97, 129.00, 128.77, 128.70, 121.28, 116.90. Data are in agreement with those previously reported.[2]

*1.3. Synthesis of 1-(4-carbamothioylphenyl) 4-octyl 2-methylenesuccinate (**8b**)*

4-OI (compound **2**, 0.82 mmol, 1 eq), synthesized as reported in literature,[3] was dissolved in dichloromethane (5 mL) under argon. Then, 4-hydroxybenzothioamide (compound **6**, 0.82 mmol, 1 eq), DMAP (0.08 mmol, 0.1 eq), and DCC (1.21 mmol, 1.48 eq) were added to the solution at 0 °C. The reaction mixture was left under argon atmosphere at room temperature overnight. The obtained yellow oil was purified by column chromatography using hexane/EtOAc (7:3) mixture as eluent. Yellow solid (24%): mp 49–52 °C; IR (KBr) cm^{-1} 2913, 1714, 1634, 1598, 1434, 885, 722, 622, 567, 474, 455; ^{1}H NMR (500 MHz, DMSO–d_6) δ 9.89 (s, 1H, thioamide), 9.52 (s. 1H, thioamide), 7.96 (d, J = 9.0 Hz, 2H, aromatic), 7.17 (d, J = 9.0 Hz, 2H, aromatic), 6.46 (s, 1H, COCCH_AH$_B$), 6.06 (s, 1H, COC=CH$_A$$H_B$), 4.05 (d, J = 6.5 Hz, 2H, OCH_2CH$_2$(CH$_2$)$_5$CH$_3$), 3.51 (s, 2H, COCH_2C=CH$_2$), 1.57–1.52 (m, 2H, OCH$_2$CH_2(CH$_2$)$_5$CH$_3$), 1.28–1.20 (m, 10H, OCH$_2$CH$_2$(CH_2)$_5$CH$_3$), 0.83 (t, J = 7.0 Hz, 3H, OCH$_2$CH$_2$(CH$_2$)$_5$CH_3); ^{13}C NMR (125 MHz, DMSO–d_6) δ 198.91, 170.27, 164.20, 152.53, 137.11, 133.26, 131.00, 128.84, 120.96, 64.40, 37.16, 31.17, 28.57, 28.10, 25.31,

22.05, 13.93. Anal. Calcd. for $C_{20}H_{27}NO_4S$: C, 63.63; H, 7.21; N, 3.71; Found: C, 63.47; H, 7.19; N, 3.72.

*1.4. Synthesis of 4-carbamothioylphenyl methyl fumarate (**8c**)*

The commercially available monomethyl fumarate (compound **3**, 1.15 mmol, 1 eq) was dissolved in dry DMF (6 mL) and the temperature was lowered to 0 °C with an ice bath. EDC hydrochloride (1.72 mmol, 1.5 eq), HOBt (1.72 mmol, 1.5 eq), DMAP (0.12 mmol, 0.1 eq), and 4-hydroxybenzothioamide (compound **6**, 1.15 mmol, 1 eq) were subsequently added to the reaction mixture. The solution was left stirring at room temperature under argon atmosphere. After 48 hours, the solvent was removed under vacuum. The obtained crude material was purified by column chromatography using EtOAc/hexane (5:5) mixture as eluent. Yellow solid (28%): mp 182–185 °C; IR (KBr) cm^{-1} 3367, 3188, 1726, 1628, 1508, 1439, 1314, 1263, 1169, 977, 890, 859, 768; ^1H NMR (500 MHz, DMSO–d_6) δ 9.91 (s, 1H, thioamide), 9.54 (s, 1H, thioamide), 7.96 (d, J = 8.5 Hz, 2H, aromatic), 7.28 (d, J = 8.5 Hz, 2H, aromatic), 7.00 (s, 2H, C*H*=C*H*), 3.79 (s, 3H, OCH_3); ^{13}C NMR (125 MHz, DMSO–d_6) δ 198.95, 164.65, 162.80, 152.10, 137.46, 134.56, 132.35, 128.80, 121.06, 52.44. Anal. Calcd. for $C_{12}H_{11}NO_4S$: C, 54.33; H, 4.18; N, 5.28. Found: C, 54.21; H, 4.17; N, 5.30.

1.5. General procedure for the synthesis of compounds 8d–f

In a round bottom flask, the appropriate carboxylic acid **10a**–**c** (1 eq) was dissolved in 4 mL of anhydrous DMF under an argon atmosphere. The solution was cooled with an ice bath to 0 °C, and EDC hydrochloride (1.5 eq), HOBt (1.5 eq) and 4-hydroxythiobenzamide (compound **6**, 2 eq) were subsequently added, waiting 10 minutes in between each addition, to the cooled stirred solution. Subsequently, the ice bath was removed, and the solution was left under argon atmosphere, at room temperature overnight. Then, the reaction crude was diluted with EtOAc and washed three times with distilled water. The organic phase was dried with Na_2SO_4, filtered, and evaporated under vacuum to obtain a yellow solid. Thus, the crude was washed with diethyl ether and the precipitate was filtered under vacuum with a Buchner filter (porosity grade 3).

*1.5.1. 4-Carbamothioylphenyl (E)-4-oxo-4-(phenylamino)but-2-enoate (**8d**)*

The title compound was obtained using 0.58 mmol of (*E*)-4-oxo-4-(phenylamino)but-2-enoic acid (*10a*), 0.87 mmol of EDC hydrochloride, 0.87 mmol of

HOBt, and 1.16 mmol of compound **6**. Yellowish solid (47%): mp 212–214 °C; IR (KBr) cm^{-1} 3416, 3342, 3256, 3142, 1738, 1679, 1650, 1620, 1599, 1546, 1503, 1492, 1444, 1427, 1338, 1305, 1201, 1166, 1151, 990, 903, 865, 770, 710, 551; ^{1}H NMR (500 MHz, DMSO–d_6) δ 10.64 (s, 1H, NH), 9.92 (s, 1H, thioamide), 9.55 (s, 1H, thioamide), 7.97 (d, J = 9.0 Hz, 2H, aromatic), 7.71 (d, J = 7.5 Hz, 2H, aromatic), 7.43 (d, J = 15.0 Hz, 1H, C*H*=CHCOO), 7.37 (t, J = 7.5 Hz, 2H, aromatic), 7.30 (d, J = 9.0 Hz, 2H, aromatic), 7.13 (t, J = 7.5 Hz, 1H, aromatic), 6.94 (d, J = 15.0 Hz, 1H, CH=C*H*COO); ^{13}C NMR (125 MHz, DMSO–d_6) δ 198.99, 163.44, 160.96, 152.25, 139.58, 138.46, 137.41, 128.95, 128.83, 121.16, 119.45. Anal. Calcd. for $C_{17}H_{14}N_2O_3S$: C, 62.56; H, 4.32; N, 8.58; Found: C, 62.67; H, 4.32; N, 8.59.

*1.5.2. 4-Carbamothioylphenyl (E)-4-(benzylamino)-4-oxobut-2-enoate (**8e**)*

The title compound was obtained using 0.29 mmol of (*E*)-4-(benzylamino)-4-oxobut-2-enoic acid (**10b**), 0.43 mmol of EDC hydrochloride, 0.43 mmol of HOBt, and 0.58 mmol of compound **6**. Yellowish solid (45%): mp 182–184 °C; IR (KBr) cm^{-1} 3305, 3157, 1730, 1663, 1634, 1548, 1503, 1426, 1324, 1290, 1212, 1170, 1146, 994, 894, 848, 736, 680; ^{1}H NMR (500 MHz, DMSO–d_6) δ 9.91 (s, 1H, thioamide), 9.54 (s, 1H, thioamide), 9.12 (t, J = 5.5 Hz, 1H, NH), 7.96 (d, J = 8.5 Hz, 2H, aromatic), 7.37–7.26 (m, 8H, aromatic + C*H*=CHCOO), 6.84 (d, J = 15 Hz, 1H,CH=C*H*COO), 4.46 (d, J = 5.5 Hz, 2H, CH_2); ^{13}C NMR (125 MHz, DMSO–d_6) δ 198.99, 163.51, 162.53, 152.26, 139.23, 138.54, 137.35, 128.80, 128.41, 127.65, 127.40, 127.05, 121.14, 42.55. Anal. Calcd. for $C_{18}H_{16}N_2O_3S$: C, 63.51; H, 4.74; N, 8.23; Found: C, 63.59; H, 4.74; N, 8.22.

*1.5.3. 4-Carbamothioylphenyl (E)4-((4-chlorobenzyl)amino)-4-oxobut-2-enoate (**8f**)*

The title compound was obtained using 1.03 mmol of (*E*)-4-((4-chlorobenzyl)amino)-4-oxobut-2-enoic acid (**10c**), 1.54 mmol of EDC hydrochloride, 1.54 mmol of HOBt, and 2.06 mmol of compound **6**. Yellowish solid (47%): mp 188–190 °C; IR (KBr) cm^{-1} 3302, 3154, 1729, 1664, 1633, 1537, 1503, 1492, 1427, 1320, 1291, 1212, 1170, 1147, 1016, 896, 848, 807, 536; ^{1}H NMR (500 MHz, DMSO–d_6) δ 9.90 (s, 1H, thioamide), 9.53 (s, 1H, thioamide), 9.15 (t, J = 6.0 Hz, 1H, NH), 7.95 (d, J = 9.0 Hz, 2H, aromatic), 7.41 (d, J = 8.5 Hz, 2H, aromatic), 7.32 (d, J = 8.0 Hz, 2H, aromatic), 7.27–7.24 (m, 3H, aromatic + C*H*=CHCOO), 6.84 (d, J = 15.0 Hz, 1H,CH=C*H*COO), 4.41 (d, J = 6.0 Hz, 2H, CH_2); ^{13}C NMR (125 MHz, DMSO–d_6) δ 199.04, 163.52, 162.69, 152.28, 139.08, 137.68, 137.40, 131.64, 129.28, 128.84, 128.39,

127.81, 121.18, 41.89. Anal. Calcd. for $C_{18}H_{15}ClN_2O_3S$: C, 57.68; H, 4.03; N, 7.47; Found: C, 57.76; H, 4.03; N, 7.46.

*1.6. Synthesis of (E)-4-(3-oxo-3-phenethoxyprop-1-en-1-yl)-1,2-phenylene bis(4-carbamothioylbenzoate) (**8g**)*

In a flamed round-bottom flask, compound **7** (0.83 mmol, 3 eq) was dissolved in dry DMF (5 mL) under an argon atmosphere. The solution was cooled to 0 °C with an ice bath and EDC hydrochloride (0.69 mmol, 2.5 eq) was added followed by HOBt (0.69 mmol, 2.5 eq) after 15 minutes. Finally, after 15 additional minutes from HOBt addition, compound **4** (CAPE, 0.276 mmol, 1 eq) was added. The reaction was allowed to stir at room temperature for 5 hours. Deionized water was poured and the mixture extracted three times with EtOAc; the reunited organic phases were washed with brine, dried with Na_2SO_4, filtered and evaporated. The crude was purified by flash chromatography eluting with a gradient mixture of hexane and EtOAc. Yellow solid (65%): mp 200-201 °C; IR (KBr) cm^{-1} 3366, 3286, 3167, 1747, 1697, 1634, 1498, 1427, 1326, 1250, 1174, 1115, 1068, 1056, 1013, 897, 859, 697; ^{1}H NMR (500 MHz, DMSO–d_6) δ 10.09 (s, 2H, thioamide), 9.67 (s, 2H, thioamide), 8.03–8.00 (m, 5H, aromatic), 7.90 (t, *J* = 8.5 Hz, 4H, aromatic), 7.81 (d, *J* = 8.5 Hz, 1H, aromatic), 7.70 (d, *J* = 16.0 Hz, 1H, C*H*=CHCO), 7.61 (d, *J* = 8.5 Hz, 1H, aromatic), 7.34–7.28 (m, 4H, aromatic), 7.24–7.22 (m, 1H, aromatic), 6.72 (d, *J* = 16.0 Hz, 1H, CH=C*H*CO), 4.39 (t, *J* = 6.5 Hz, 2H, OCH_2CH$_2$), 2.98 (t, *J* = 6.5 Hz, 2H, OCH$_2$CH_2); ^{13}C NMR (125 MHz, DMSO–d_6) δ 199.01, 165.92, 162.98, 162.83, 144.51, 143.38, 142.73, 142.27, 137.98, 133.33, 129.48, 129.32, 128.86, 128.37, 127.67, 127.63, 126.37, 124.27, 123.23, 119.42, 64.70, 34.39; Anal. Calcd. for $C_{33}H_{26}N_2O_6S_2$: C, 64.90; H, 4.29; N, 4.59. Found: C, 64.85; H, 4.30; N, 4.56.

*1.7. Synthesis of (E)-4-(3,5-dimethoxystyryl)phenyl 4-carbamothioylbenzoate (**8h**)*

In a flame-dryed round-bottom flask, compound 5 (pterostilbene, 0.219 mmol, 1 eq), compound 7 (0.33 mmol, 1.5 eq) and a catalytic amount of DMAP were suspended in 3 mL of dry CH2Cl2. The mixture was cooled to 0 °C with an ice bath and EDC hydrochloride (0.33 mmol, 1.5 eq) was added under argon. The mixture was stirred at room temperature overnight in the dark. The obtained yellow suspension was filtered under vacuum and the precipitate was discarded. Silica gel was added to the filtrate, the mixture was rotavaped in the dark and the crude purified eluting with a gradient mixture of EtOAc in hexane in the dark. Fluffy pale yellow solid (33%): mp 217–219 °C; IR

(KBr) cm^{-1} 3351, 3285, 3169, 2929, 2833, 1721, 1639, 1591, 1508, 1458, 1424, 1336, 1306, 1274, 1202, 1164, 1146, 1080, 1016, 968, 943, 909, 890, 864, 830, 772, 699; ^{1}H NMR (500 MHz, DMSO–d_6) δ 10.12 (s, 1H, thioamide), 9.74 (s, 1H, thioamide), 8.16 (d, J = 8.5 Hz, 2H, aromatic), 8.02 (d, J = 8.0 Hz, 2H, aromatic), 7.70 (d, J = 8.5 Hz, 2H, aromatic), 7.34–7.31 (m, 3H, aromatic + C*H*=CHC$_6$H$_4$OCO), 7.21 (d, J = 16.5 Hz, 1H, CH=C*H*C$_6$H$_4$OCO), 6.80 (s, 2H, aromatic), 6.43 (s, 1H, aromatic), 3.79 (s, 6H, 2×OCH$_3$); ^{13}C NMR (125 MHz, DMSO–d_6) δ 199.13, 164.03, 160.65, 149.93, 144.10, 138.97, 134.97, 130.62, 129.44, 128.80, 127.93, 127.59, 127.53, 122.15, 104.53, 99.96, 55.21; Anal. Calcd. for $C_{24}H_{21}NO_4S$: C, 68.72; H, 5.05; N, 3.34. Found: C, 68.77; H, 5.03; N, 3.36.

1.8. General procedure for the synthesis of compounds 9a–c

In a round bottom flask, monomethyl fumarate (compound **6**, 1 eq) was dissolved in 13 mL of anhydrous DMF under an argon atmosphere. The solution was cooled with an ice bath to 0 °C, and EDC hydrochloride (1.5 eq), HOBt (1.5 eq) and the appropriate amine (2 eq) were subsequently added, waiting 15 minutes between each addition to the cooled stirred solution. Then, the ice bath was removed and the solution was left under stirring at room temperature overnight under argon atmosphere. Afterwards, the reaction crude was diluted with dichloromethane and washed one time with HCl 0.1 M, once with saturated $NaHCO_3$, once with water, and once with brine. Subsequently, the organic phase was dried with Na_2SO_4, filtered, and evaporated under vacuum. The crude was then solubilized in a minimum quantity of dichloromethane, and hexane was added to precipitate the final product. The precipitate was filtered under vacuum with a Buchner filter (porosity grade 3), washing it with a 1:9 dichloromethane/hexane mixture.

*1.8.1. Methyl (E)-4-oxo-4-(phenylamino)but-2-enoate (**9a**)*

The title compound was obtained using 2.69 mmol of compound **3**, 4.03 mmol of EDC hydrochloride, 4.03 mmol of HOBt, and 5.38 mmol of aniline. Yellow solid (49%): mp 79–81 °C; IR (KBr) cm^{-1} 3283, 3080, 2954, 1731, 1667, 1207, 1166, 1332, 1307, 1184, 1177, 1102, 933, 560; ^{1}H NMR (200 MHz, DMSO–d_6) δ 10.57 (s, 1H, NH), 7.68 (d, J = 10.0 Hz, 2H, aromatic), 7.35 (t, J = 6.0 Hz, 2H, aromatic), 7.24 (d, J = 16.0 Hz, 1H, C*H*=CHCOO), 7.11 (t, J = 6.0 Hz, 1H, aromatic), 6.73 (d, J = 16 Hz, 1H, CH=C*H*COO), 3.76 (s, 3H, CH$_3$). Anal. Calcd. for $C_{11}H_{11}NO_3$: C, 64.38; H, 5.40; N, 6.83; Found: C, 64.17; H, 5.38; N, 6.82.

*1.8.2. Methyl (E)-4-(benzylamino)-4-oxobut-2-enoate (**9b**)*

The title compound was obtained using 2.69 mmol of compound **3**, 4.03 mmol of EDC hydrochloride, 4.03 mmol of HOBt, and 5.38 mmol of benzylamine. White solid (28%): mp 91–93 °C; IR (KBr) cm^{-1} 3286, 2951, 1700, 1635, 1332, 1302, 1169, 1153, 1112, 923, 568; ^{1}H NMR (200 MHz, DMSO–d_6) δ 9.06 (t, *J* = 6.0 Hz, 1H, NH), 7.44–7.18 (m, 5H aromatic), 7.08 (d, *J* = 16.0 Hz, 1H C*H*=CHCOO), 6.63 (d, *J* = 16.0 Hz, 1H, CH=C*H*COO), 4.39 (d, *J* = 6.0 Hz, 2H, CH_2), 3.73 (s, 3H, CH_3). Anal. Calcd. for $C_{12}H_{13}NO_3$: C, 65.74; H, 5.98; N, 6.39; Found: C, 65.78; H, 5.99; N, 6.38.

*1.8.3. Methyl (E)-4-((4-chlorobenzyl)amino)-4-oxobut-2-enoate (**9c**)*

The title compound was obtained using 2.46 mmol of compound **3**, 3.69 mmol of EDC hydrochloride, 3.69 mmol of HOBt, and 4.92 mmol of 4-chlorobenzylamine. White solid (66%): mp 121.5–122 °C; IR (KBr) cm^{-1} 3311, 1708, 1630, 1545, 1492, 1.415, 1332, 1307, 1184, 1177, 1102, 933, 560; ^{1}H NMR (200 MHz, DMSO–d_6) δ 9.09 (t, *J* = 6.0 Hz, 1H, NH), 7.42–7.27 (m, 4H, aromatic), 7.06 (d, *J* = 16.0 Hz, 1H, C*H*=CHCOO), 6.63 (d, *J* = 16.0 Hz, 1H, CH=C*H*COO), 4.38 (d, *J* = 6.0 Hz, 2H, CH_2), 3.73 (s, 3H, CH_3). Anal. Calcd. for $C_{12}H_{12}ClNO_3$: C, 56.82; H, 4.77; N, 5.52; Found: C, 56.74; H, 4.76; N, 5.53.

1.9. General procedure for the synthesis of compounds 10a–c

In a round bottom flask, the proper monomethyl fumarate derivative **9a**–**c** (1 eq) was dissolved in 2.5 mL of CH_3OH. $LiOH{\cdot}H_2O$ (3 eq) was dissolved in distilled water to obtain a 1 M solution and added to the reaction, which was stirred for 2 h at room temperature. Then, CH_3OH was evaporated under vacuum, and the remaining water solution was treated with 0.1 M HCl dropwise until a latescent precipitate was formed. Subsequently, EtOAc was added, and the suspension was transferred to a separator funnel. The aqueous phase was extracted three times with EtOAc; the reunited organic phases were dried with Na_2SO_4, filtered, and evaporated under vacuum.

*1.9.1. (E)-4-Oxo-4-(phenylamino)but-2-enoic acid (**10a**)*

The title compound was obtained using 1.13 mmol of methyl (*E*)-4-oxo-4-(phenylamino)but-2-enoate (**9a**) and 3.39 mmol of $LiOH{\cdot}H_2O$. White solid (86%): mp 195–196 °C; IR (KBr) cm^{-1} 3272, 3072, 2884, 1695, 1619; 1357, 1322, 1184, 1168, 1099, 929, 545; ^{1}H NMR (200 MHz, DMSO–d_6) δ 10.52 (s, 1H, NH), 7.68 (d, *J* = 8.0 Hz, 2H, aromatic), 7.35 (t, *J* = 8.0 Hz, 2H, aromatic), 7.19–7.07 (m, 2H, aromatic+ C*H*=CHCOO),

6.66 (d, J = 16.0 Hz, 1H, CH=CHCOO). Anal. Calcd. for $C_{10}H_9NO_3$: C, 62.82; H, 4.75; N, 7.33; Found: C, 62.74; H, 4.76; N, 7.33.

*1.9.2. (E)-4-(Benzylamino)-4-oxobut-2-enoic acid (**10b**)*

The title compound was obtained using 0.71 mmol of methyl (*E*)-4-(benzylamino)-4-oxobut-2-enoate (**9b**) and 2.13 mmol of $LiOH{\cdot}H_2O$. White solid (87%): mp 125–127 °C; IR (KBr) cm^{-1} 3248, 3065, 2947, 1697, 1629, 1339, 1301, 1178, 1100, 931, 566; ^1H NMR (200 MHz, DMSO–d_6) δ 9.01 (t, J = 6.0 Hz 1H, NH), 7.38–7.25 (m, 5H, aromatic), 6.99 (d, J = 16.0 Hz, 1H, CH=CHCOO), 6.56 (d, J = 16.0 Hz, 1H, CH=CHCOO), 4.38 (d, J = 6.0 Hz, 2H, CH_2). Anal. Calcd. for $C_{11}H_{11}NO_3$: C, 64.38; H, 5.40; N, 6.83; Found: C, 64.24; H, 5.39; N, 6.82.

*1.9.3. (E)-4-((4-Chlorobenzyl)amino)-4-oxobut-2-enoic acid (**10c**)*

The title compound was obtained using 1.51 mmol of methyl (*E*)-4-((4-chlorobenzyl)amino)-4-oxobut-2-enoate (**9c**) and 4.53 mmol of $LiOH{\cdot}H_2O$. White solid (98%): mp 232–234 °C; IR (KBr) cm^{-1}: 3286, 3085, 1689, 1652, 1556, 1492, 1414, 1332, 1265, 1188, 1101, 1036, 1016, 983, 932, 846, 803, 725, 569, 410; ^1H NMR (200 MHz, DMSO–d_6) δ 9.05 (t, J = 6.0 Hz, 1H, NH), 7.40 (d, J = 8.0 Hz, 2H, aromatic), 7.30 (d, J = 8.0 Hz, 2H, aromatic), 6.98 (d, J = 16.0 Hz, 1H, CH=CHCOO), 6.56 (d, J = 16.0 Hz, 1H, CH=CHCOO), 4.37 (d, J = 6.0 Hz, 2H, CH_2). Anal. Calcd. for $C_{11}H_{10}ClNO_3$: C, 55.13; H, 4.21; N, 5.84; Found: C, 55.07; H, 4.22; N, 5.83.

2. Pharmacology

2.1. Amperometric measurement of H_2S release

The H_2S-releasing properties of the tested compounds were evaluated by a cell-free amperometric approach, using H_2S-selective electrodes connected to the Apollo-4000 Free Radical Analyzer (WPI, FL, USA). The electrode was immersed in 2 mL PBS buffer at room temperature and pH 7.4, until a stable baseline was reached. Then, the tested compounds were incubated in the PBS buffer, in the absence or presence of L-Cysteine 4 mM, at a final concentration of 100 μM and 1% of DMSO. The release of H_2S was observed for 30 min. L-cysteine was added to reproduce the endogenous presence of organic thiols. NaHS 1 μM incubated in a PBS buffer at pH 4.0 was used as reference H_2S-donor.

2.2. Cell line

HASMCs (Life Technologies, USA) were cultured in Human Vascular Smooth Muscle Cell Basal Medium (Life technologies, USA) with the addition of Smooth Muscle Growth Supplement (SMGS, Life Technologies, USA), 1% of 100 units/mL penicillin and 100 mg/mL streptomycin (Sigma-Aldrich, USA) in tissue culture flasks at 37 °C in a 90% humidified atmosphere and 5% CO_2.

2.3. Intracellular release of H_2S

HASMCs were seeded on a 96-well black plate, pre-coated with gelatin from porcine skin 1% (Merck), at density of 30×10^3/well. After 24 h, the culture medium was replaced with 180 µL of standard buffer (composition: HEPES 20 mM, KCl 2 mM, NaCl 120 mM, $MgCl_2\cdot6H_2O$ 1 mM, $CaCl_2\cdot2H_2O$ 2 mM, Glucose 5 mM, pH 7.4) containing the Washington State Probe-1 (1,3′-methoxy-3-oxo-3H-spiro[isobenzofuran-1,9′-xanthen]-6′-yl-2-pyridin-2-yldisulfanyl benzoate, WSP-1) dye (Cayman Chemical), at the final concentration of 100 µM. WSP-1 is a highly sensitive probe for H_2S detection that selectively reacts with H_2S by releasing a fluorophore. HASMCs were incubated with WSP-1 at 37 °C, CO_2 (5%) for 30 min. Then, the supernatant was removed and replaced with 180 µL of standard buffer. After the assessment of the baseline fluorescence index (FI), cells were treated with vehicle (DMSO 1%, Merck), diallyldisulfide (DADS, Merck) or the tested compounds 100 µM. DADS, a natural polysulfide derived from *Alliaceae* family, was used as H_2S-donor reference drug. The increase in fluorescence (expressed as FI) corresponded to the release of intracellular H_2S by the tested compounds and was monitored every 5 min at λ_{ex} = 465 nm and λ_{em} = 515 nm for 50 min using the multiwell plate reader EnSpire (PerkinElmer).

2.4. Cell viability prevention against oxidative damage

HASMCs at 90% confluence were seeded in a clear 96-well plate at a density of 10×10^3/well. After 24 h, the culture medium was replaced with 80 µL of fresh medium, and vehicle (DMSO 1%), **8b** (3 µM and 1 µM), BTA (3 µM and 1 µM), or 4-OI, as non H_2S-donor reference, (3 µM and 1 µM) were added. After incubating 1 h, the oxidative cell damage was induced by the addition of a freshly prepared solution of H_2O_2 200 µM in culture medium. After the addition of H_2O_2, cells were incubated 2 h or 24 h depending on the protocol. At the end of the incubation period, WST-1 (Roche) was incubated in each well (1:10) at 37 °C in a CO_2 (5%) incubator for 1 h. WST-1 is a substrate of dehydrogenase enzymes, present in viable cells which convert the dye in a dark yellow

product. Cell viability was spectrophotometrically assessed at λ = 495 nm using a multiwell plate reader.

2.5. Preventive effect on ROS production against H_2O_2-induced oxidative stress

Cells at 90% confluence were seeded onto a 96-well black plate (3×10^4 cells/well) pre-coated with 1% porcine gelatin. At the end of the treatment (see previous paragraph), dihydroethidium (DHE, 10 µM, Merck) was used for the evaluation of ROS production. DHE was solubilized in DMSO, diluted in Hank's balanced salt solution and incubated at 37 °C for 30 min in the dark. During the incubation period, DHE freely permeates cell membranes and reacts with ROS forming a red fluorescent product (ethidium). Fluorescence was then evaluated using a microplate reader at λ_{ex} = 500 nm and λ_{em} = 580 nm.

2.6. Evaluation of the hyperpolarizing properties of 8b, 4-OI and BTA

HASMCs were seeded onto a 96-well black plate ($7.2x10^4$ cells/well) precoated with gelatin 1% and incubated overnight. The day after, the medium was removed and cells were incubated for 1 hour with the anionic bisoxonol dye bis(1,3-dibutylbarbituric acid) (DiBac4(3)) 2.5 µM, dissolved in PBS. A reduction in fluorescence, indicating the outward movement of the dye, represents a marker for membrane hyperpolarization. NS1619, a BKCa potassium channel activator, was used as a reference drug. After incubation, the baseline fluorescence was recorded. Then, cells were exposed to different concentrations of 4-OI and **8b** (300, 100 µM, and 1 mM), NS1619 10 µM, or vehicle (DMSO 0.5%). Fluorescence variations were monitored over a period of 35 min. The relative decrease in fluorescence was calculated using the formula (Ft − F0)/F0, where F0 indicates the baseline fluorescence and Ft indicates the fluorescence at time t following compound administration.

2.7. Animal procedures and ethical statements

All animals procedures were conducted in accordance with the guidelines of the European Community Council Directive 86-609 and in accordance with the Code of Ethics of the World Medical Association (Declaration of Helsinki, EU Directive 2010/63/EU for animal experiments). The experiments were carried out with the authorization of the Ethical Committee of the University of Pisa and of the Italian Ministry of Health (authorization number DB173.N.IXS). The animals were housed in

humidity- and temperature-controlled rooms (22 °C and 50%, respectively) with 12 h light/dark cycles, ad libitum access to water and food, and controlled environmental conditions. All possible measures were taken to reduce the number of animals used and to minimize their suffering. The animal studies were performed following the ARRIVE guidelines.[4]

2.8. Vasorelaxant effects of 8b, 4-OI, BTA in isolated rat aortic rings

Adult male normotensive Wistar rats (400–450 g) were killed by axillary exsanguination after an overdose of sodium thiopental (100 mg·kg−1 i.p.). The thoracic aorta was rapidly excised, freed of extraneous tissues and cut into 5 mm-wide rings. These aortic rings were suspended, under a preload of 2 g, in organ baths, containing 20 ml of Tyrode solution, thermostated at 37 °C, and continuously gassed with Clioxicarb (95% O_2 and 5% CO_2). An isometric transducer (Grass FTO3, USA) combined with a preamplifier (Buxco Electronics, USA) and with a software (BIOPAC Systems Inc., USA), recorded changes in tension. Endothelial layer was removed using a needle, and KCl-precontracted rings that showed an Ach- induced relaxation < 10% were considered acceptable. Each organ bath was washed and re-equilibrated with fresh Tyrode's solution for 30 min. Cumulative concentrations of 4-OI, BTA and **8b** (10^{-9}–10^{-4} M) were added to KCl-precontracted aortic rings to evaluate the direct vasorelaxant effect in absence of endothelial layer.

Furthermore, the ability to reduce norepinephrine (NA)-induced vasoconstriction of **8b** was assessed on endothelium denuded aortic rings. Specifically, 45 min after the confirmation of endothelium removal, aortic rings were contracted with 60 mM KCl. After reaching a plateau value, aortic rings were washed, and after 45 min of re-equilibration, they were pre-incubated with vehicle solution (1% DMSO) and **8b** (10^{-4} M) for 20 min. Thus, cumulative concentrations of NA (10^{-9}-10^{-6} M) were added. In order to exclude a possible toxic effect or to test the "reversibility" of the inhibitory effect of these compounds, rings were washed and, after 45 min, contracted again by KCl (60 mM).

2.9. Statistical analysis

All in vitro experiments were performed at least three times, each in triplicate. Results were expressed as mean ± SD and statistically analyzed by one-way ANOVA followed by Bonferroni's post hoc test performed using the GraphPad Prism 8 software.

Vasorelaxant and vasoconstriction values, expressed as %, were fitted and statistically analyzed using the same software. Concentration-response curves of aortas from different treatment groups were analyzed by two-way ANOVA followed by Bonferroni post-test. For all experiments, statistical significance was set at $p < 0.05$.

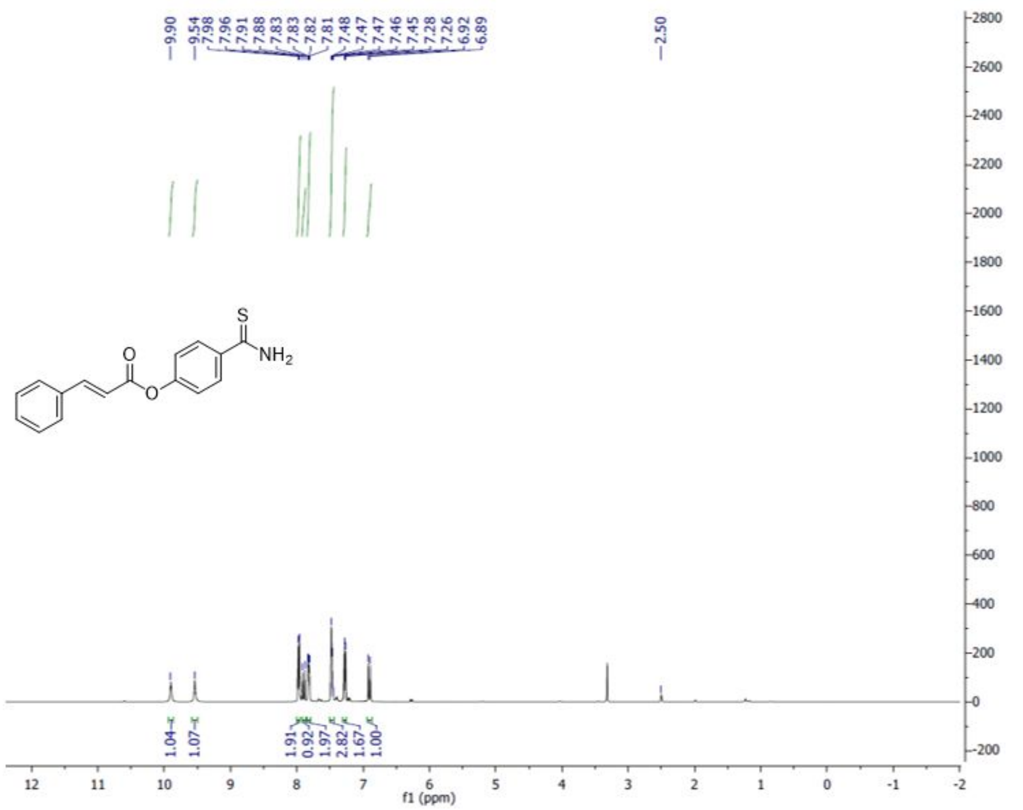

Figure S1. ^{1}H NMR (200 MHz, DMSO–d_6) of compound **8a**.

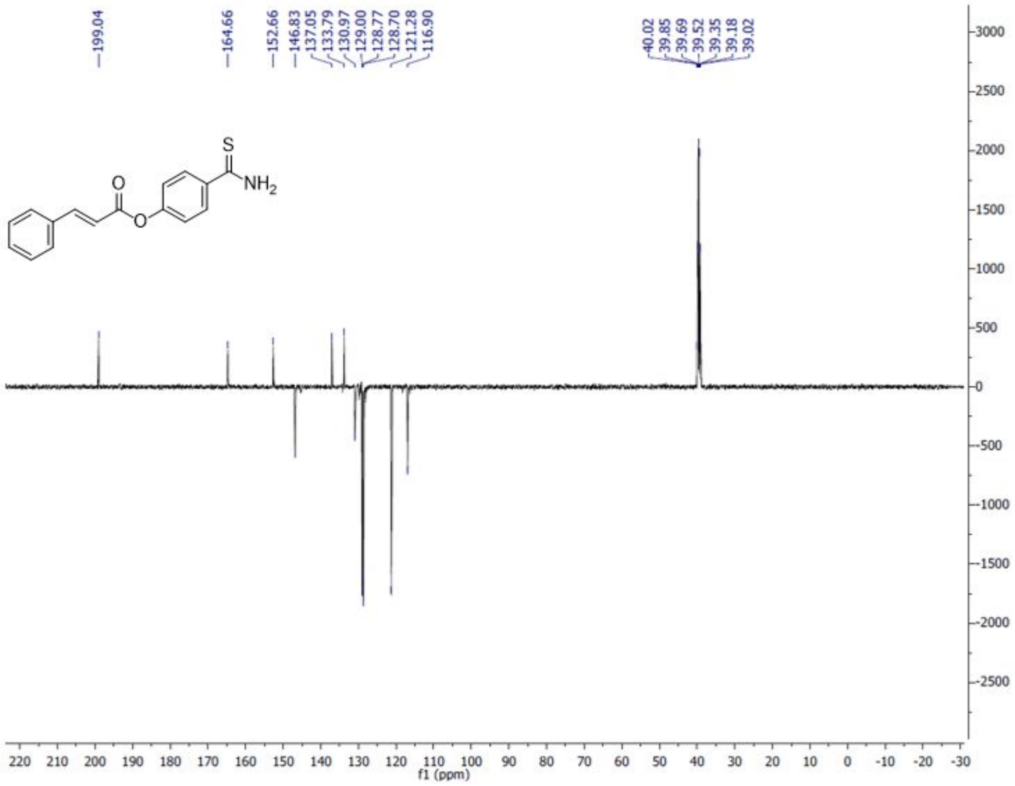

Figure S2. ^{13}C NMR (125 MHz, DMSO–d_6) of compound **8a**.

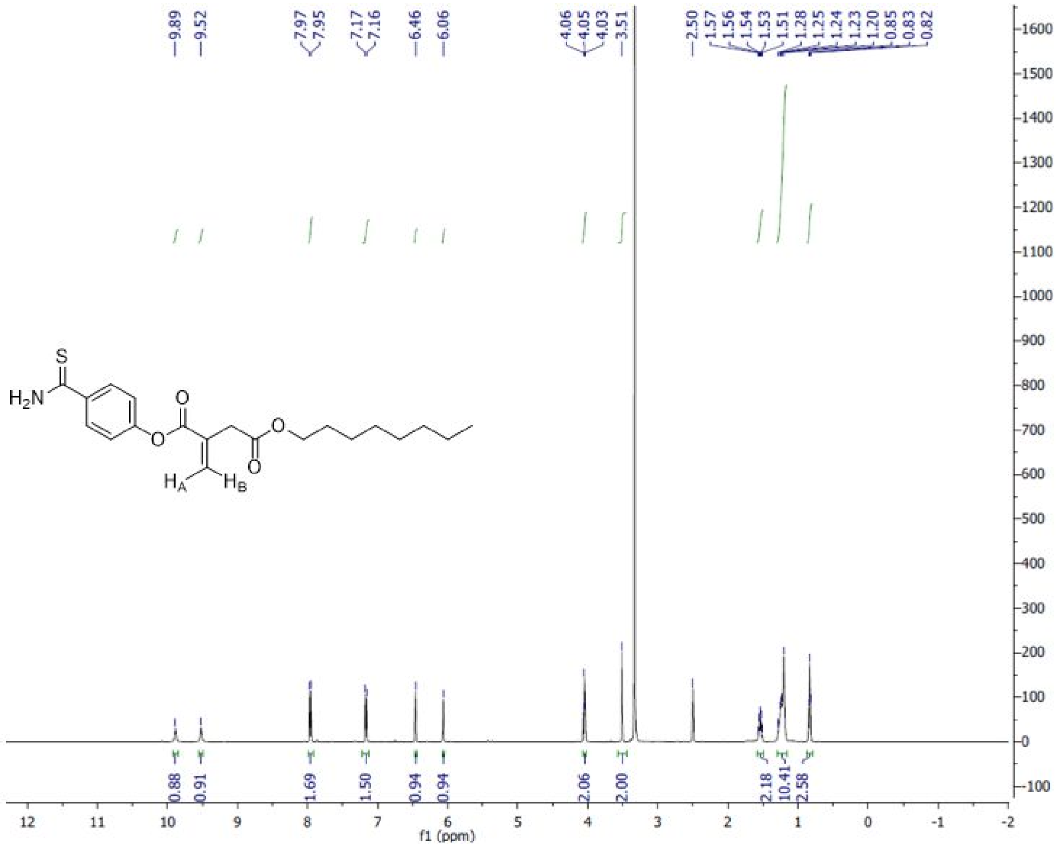

Figure S3. ^{1}H NMR (200 MHz, DMSO–d_6) of compound **8b**.

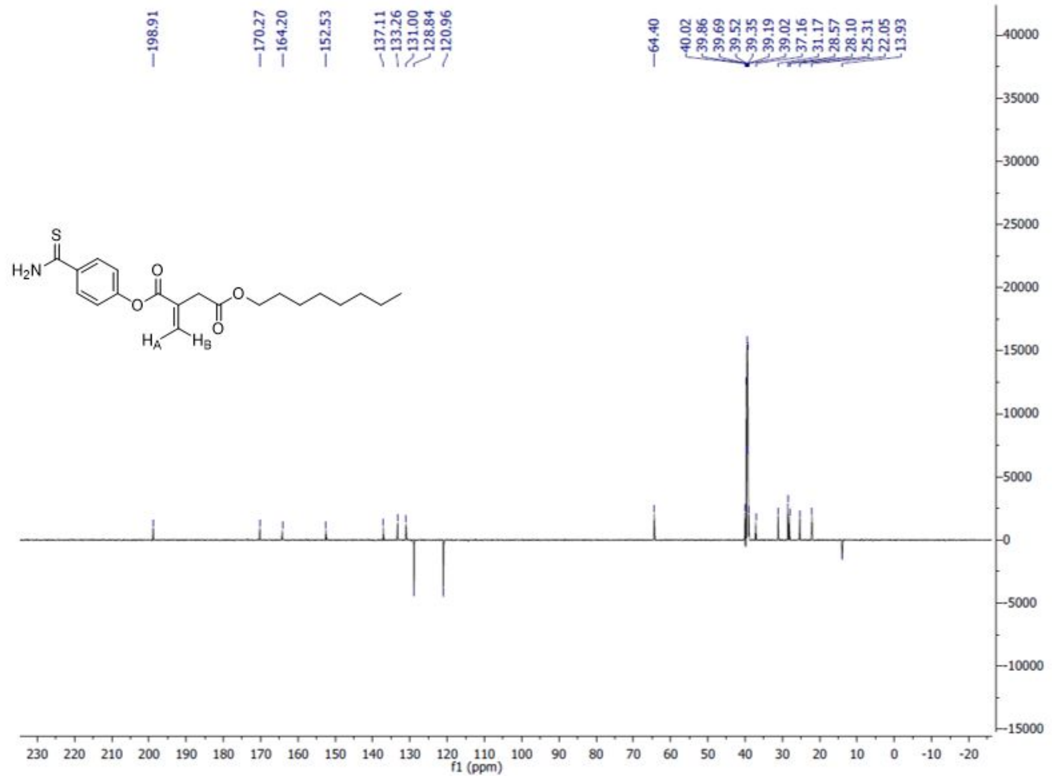

Figure S4. ^{13}C NMR (125 MHz, DMSO–d_6) of compound **8b**.

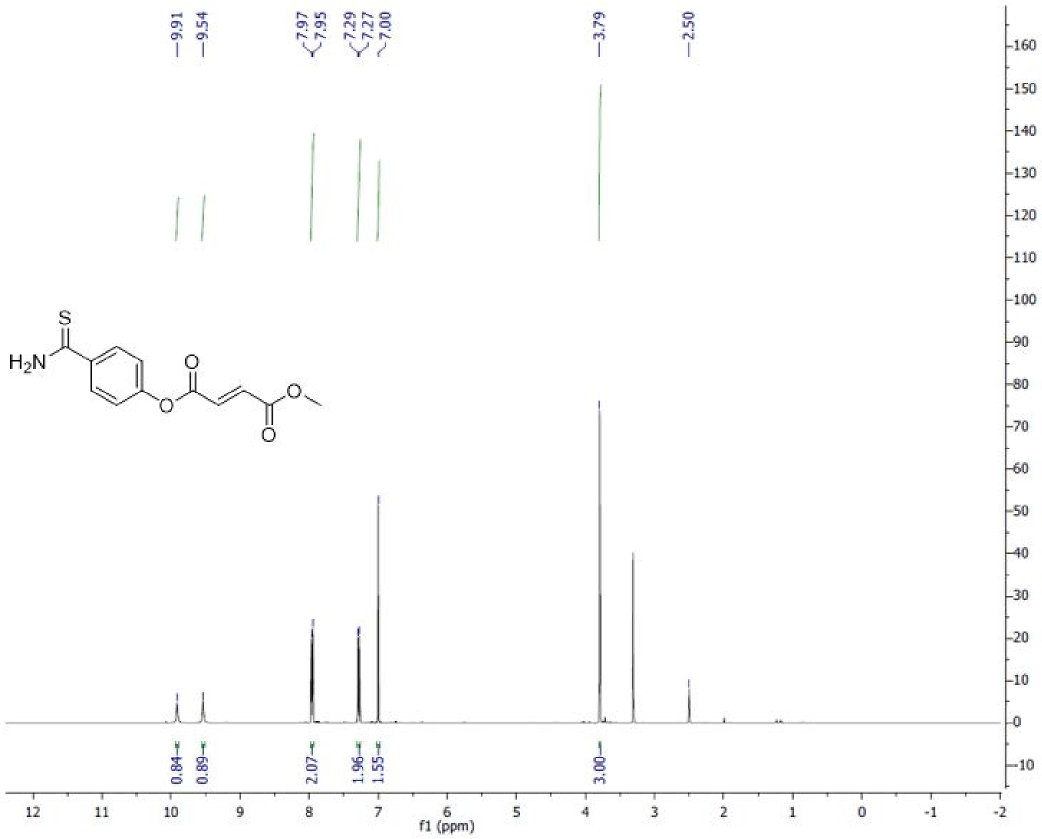

Figure S5. ^{1}H NMR (500 MHz, DMSO–d_6) of compound **8c**.

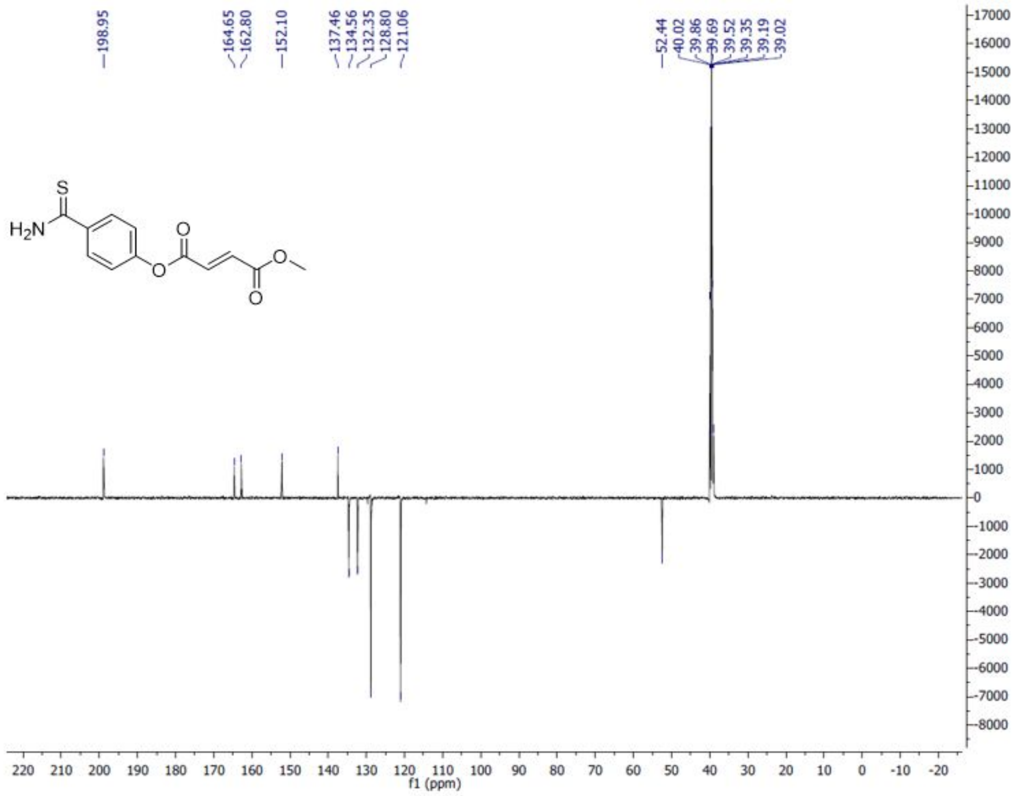

Figure S6. ^{13}C NMR (125 MHz, DMSO–d_6) of compound **8c**.

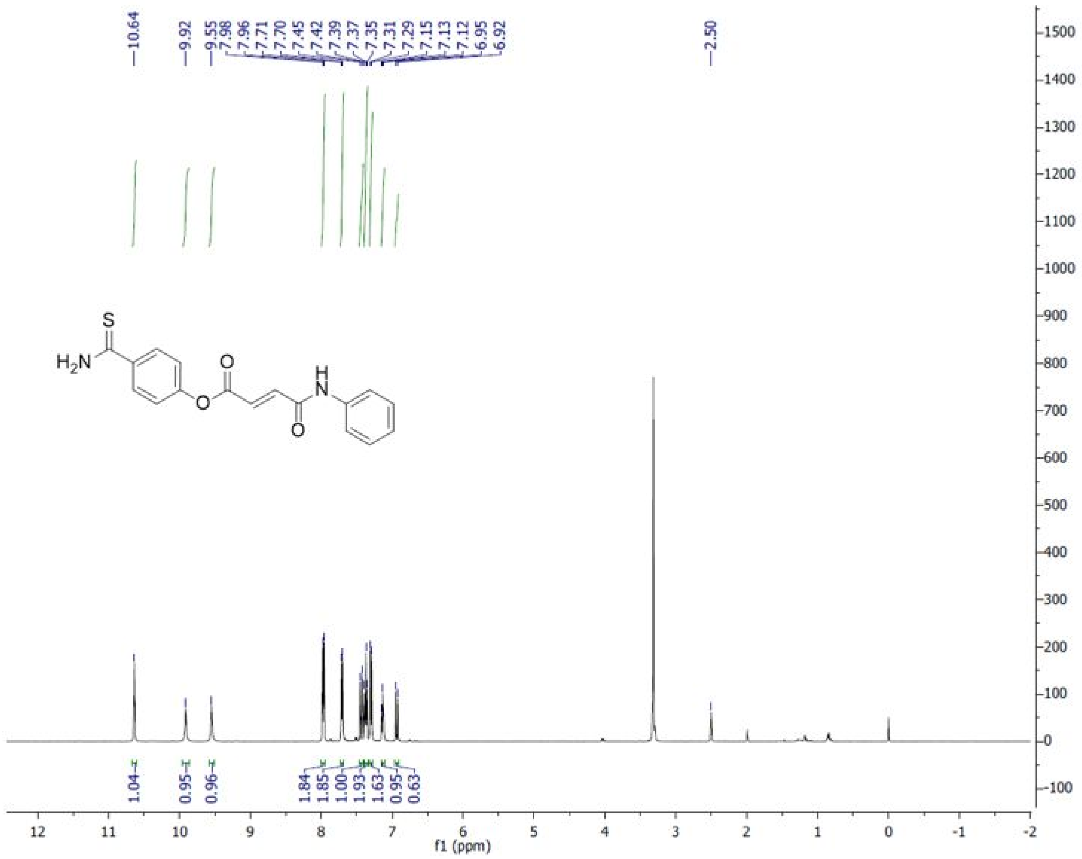

Figure S7. ^{1}H NMR (500 MHz, DMSO–d_6) of compound **8d**.

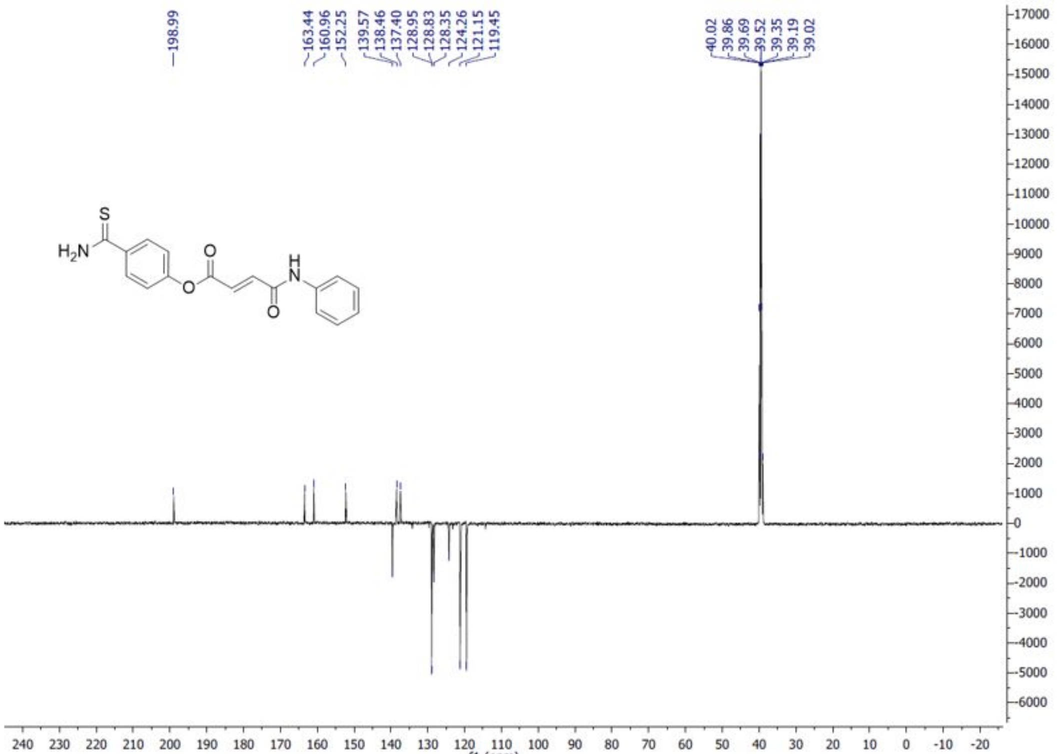

Figure S8. ^{13}C NMR (125 MHz, DMSO–d_6) of compound **8d**.

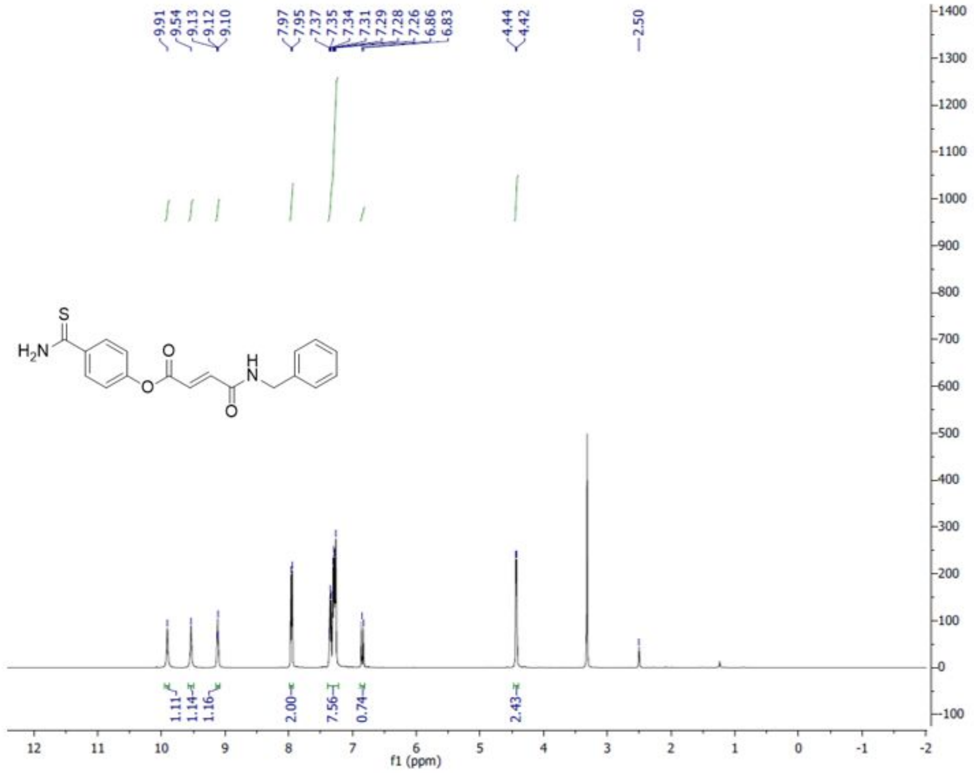

Figure S9. ^{1}H NMR (500 MHz, DMSO–d_6) of compound **8e**.

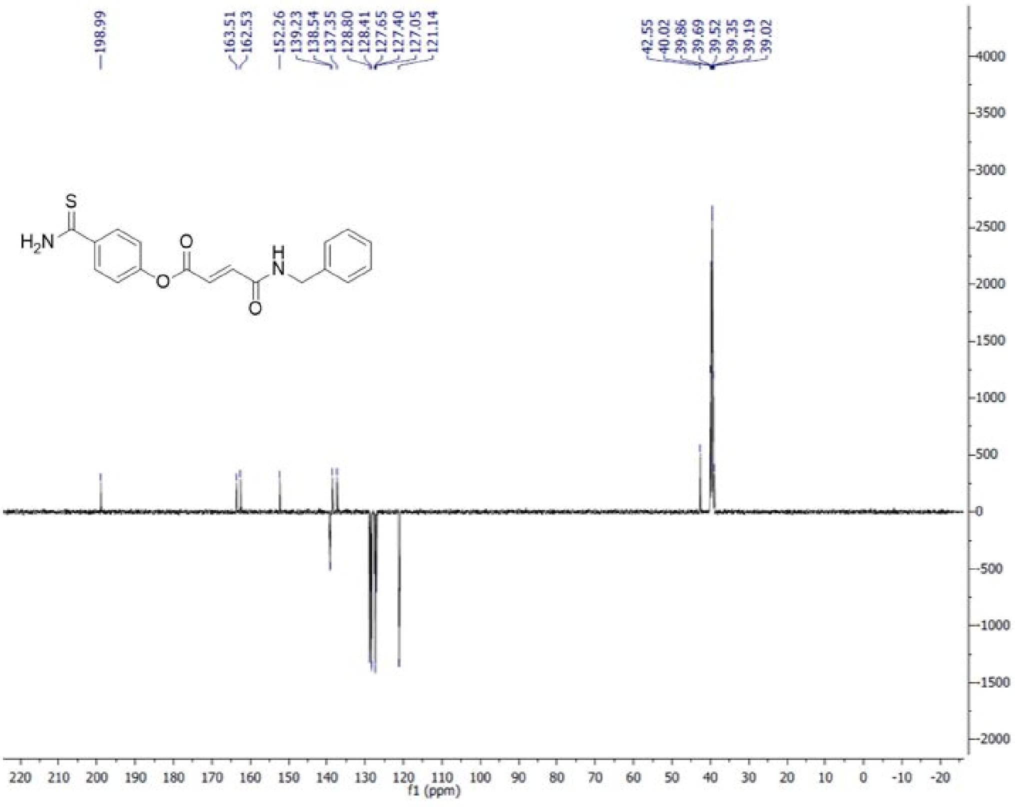

Figure S10. ^{13}C NMR (125 MHz, DMSO–d_6) of compound **8e**.

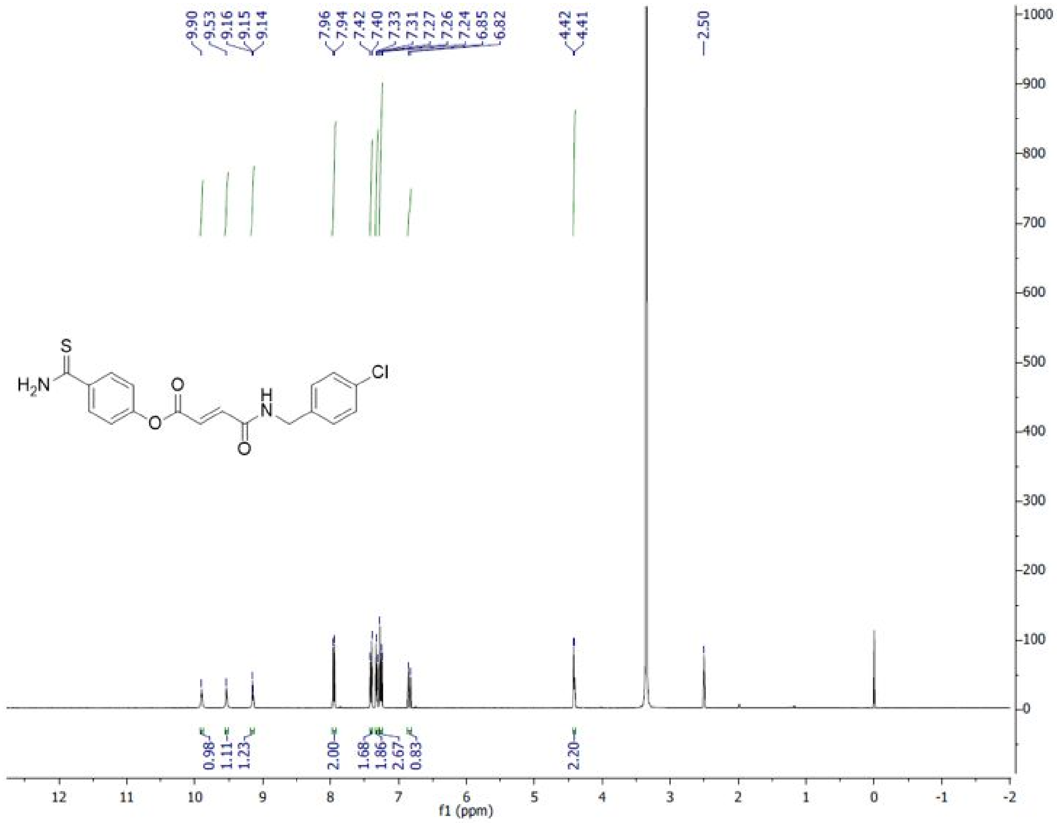

Figure S11. ^{1}H NMR (500 MHz, DMSO–d_6) of compound **8f**.

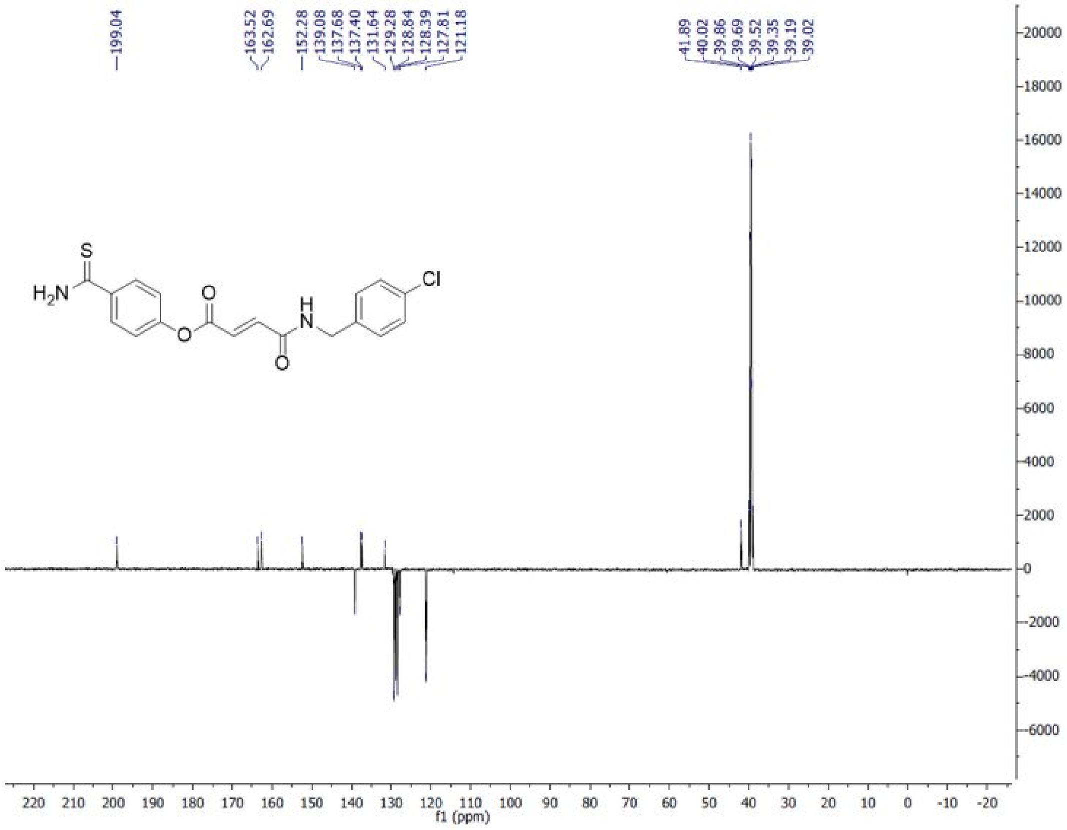

Figure S12. ^{13}C NMR (125 MHz, DMSO–d_6) of compound **8f**.

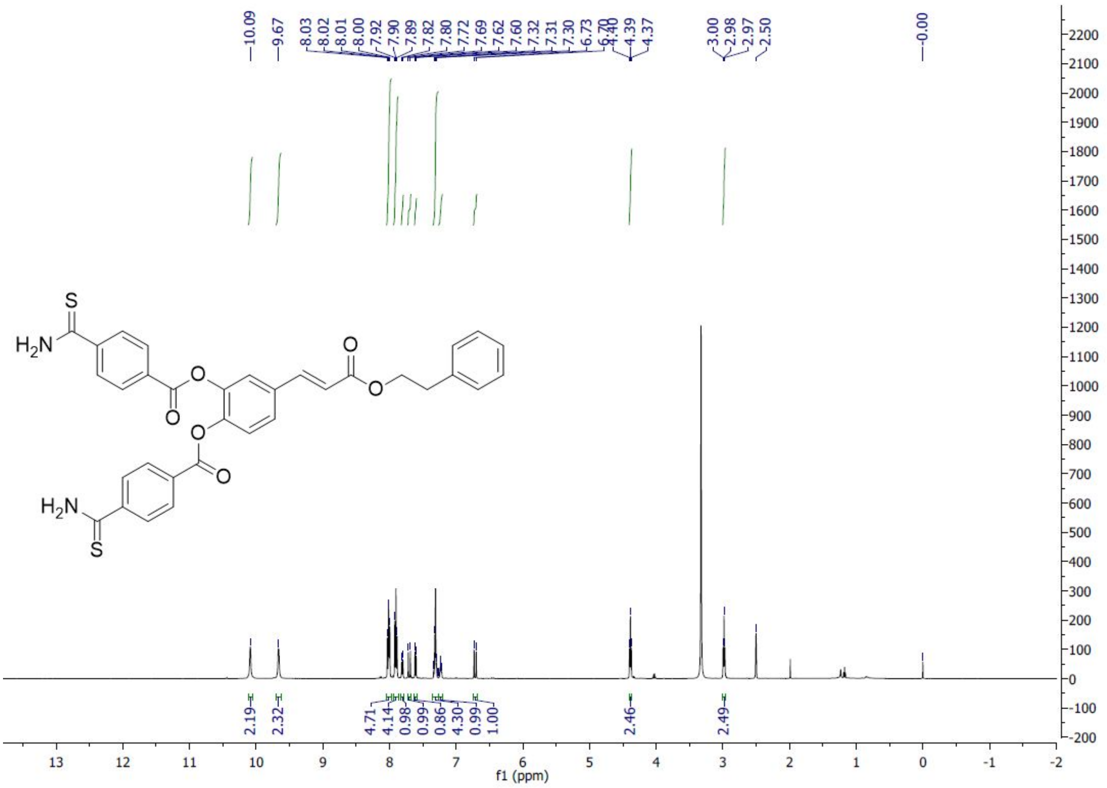

Figure S13. ^{1}H NMR (500 MHz, DMSO–d_6) of compound **8g**.

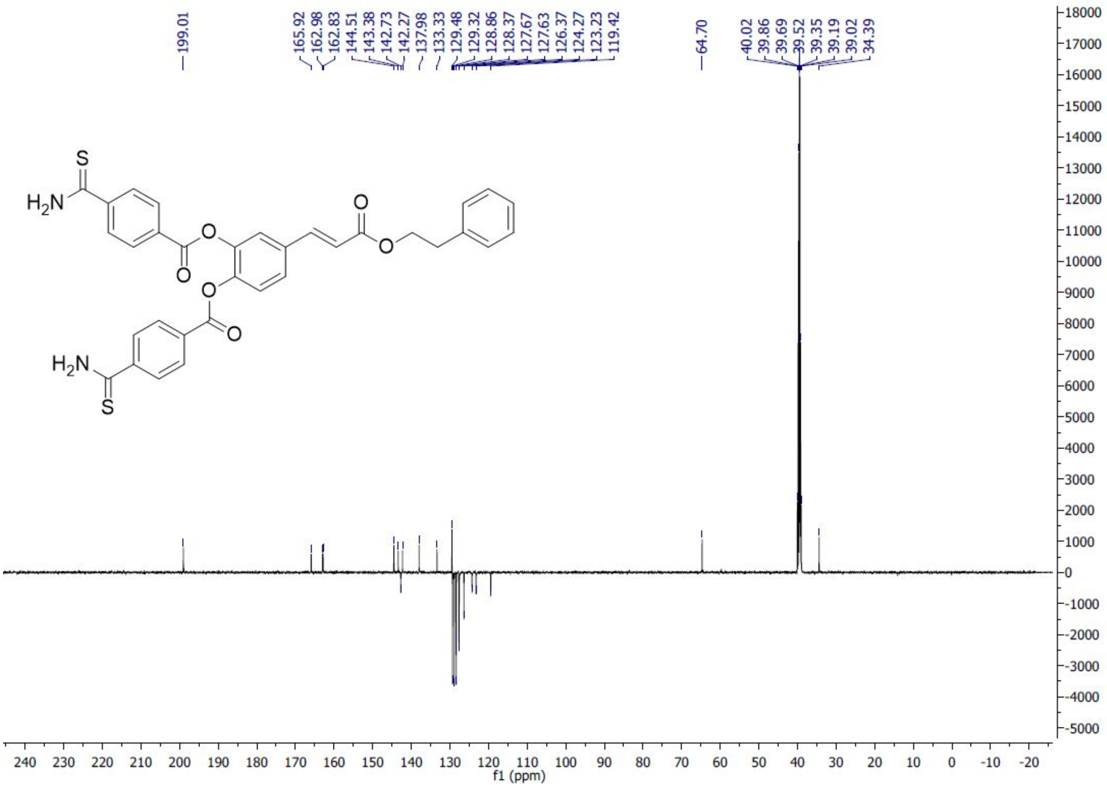

Figure S14. ^{13}C NMR (125 MHz, DMSO–d_6) of compound **8g**.

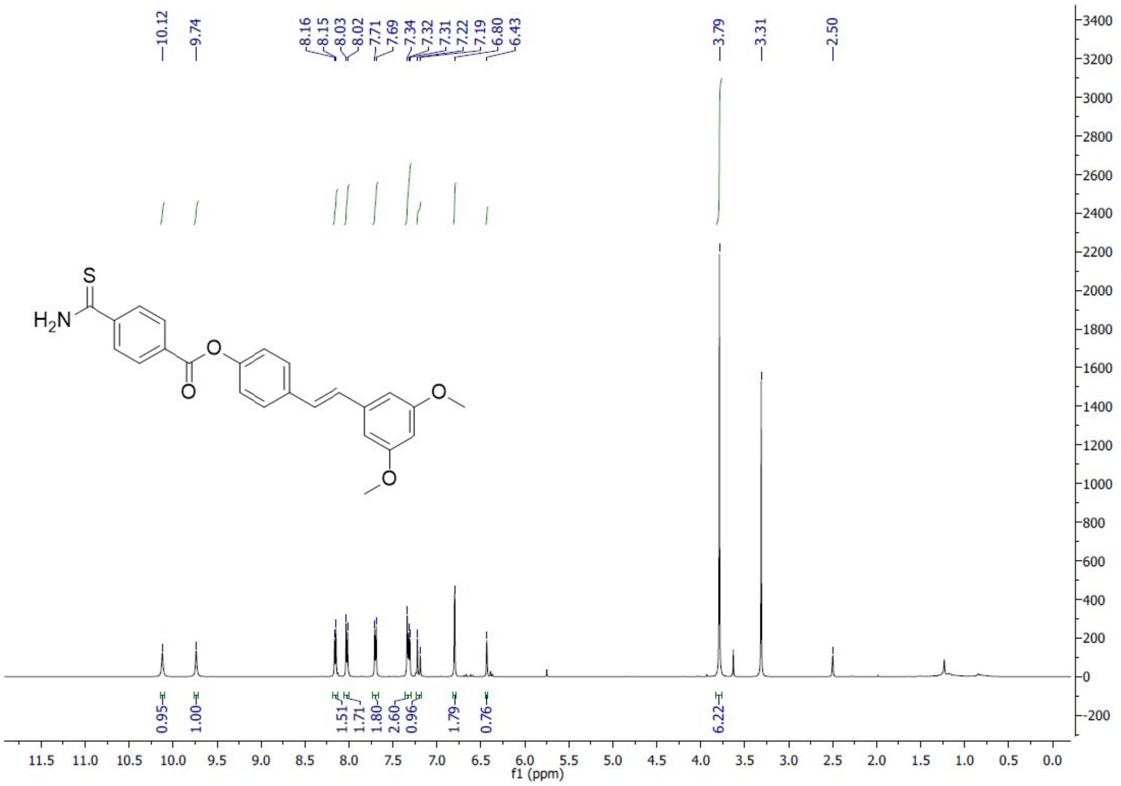

Figure S15. ^{1}H NMR (500 MHz, DMSO–d_6) of compound **8h**.

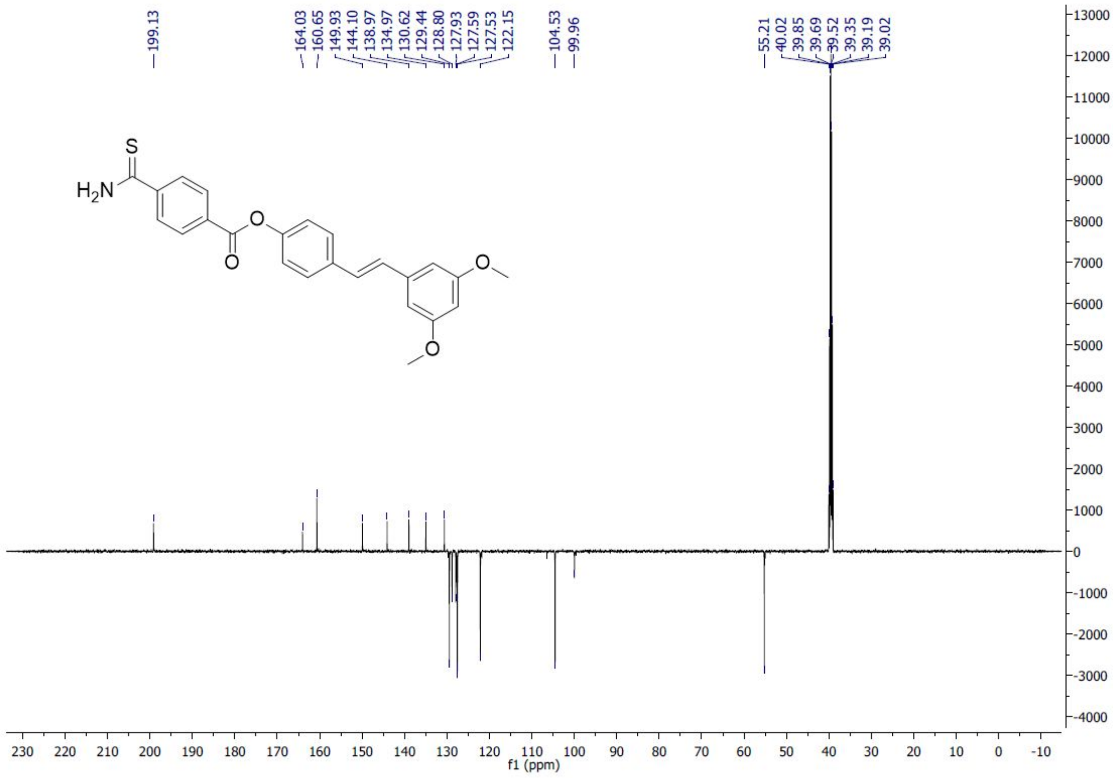

Figure S16. ^{13}C NMR (125 MHz, DMSO–d_6) of compound **8h**.

Table S1. In silico ADMET assesment for compounds **8a–h**.

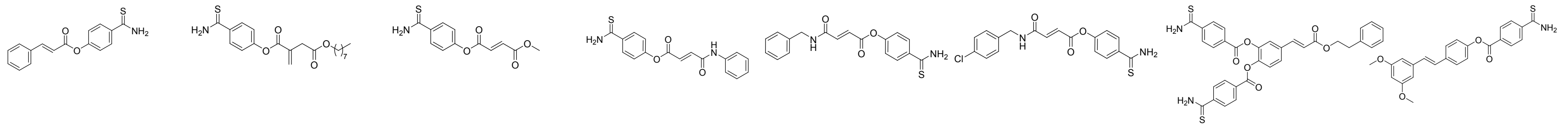

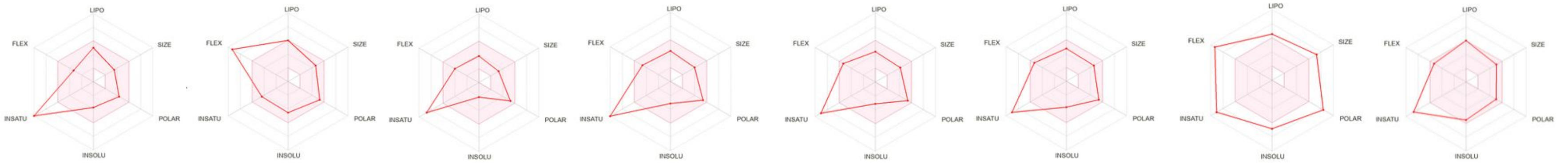

**Compound**	**8a**	**8b**	**8c**	**8d**	**8e**	**8f**	**8g**	**8h**
**Formula**	$C_{16}H_{13}NO_2S$	$C_{20}H_{27}NO_4S$	$C_{12}H_{11}NO_4S$	$C_{17}H_{14}N_2O_3S$	$C_{18}H_{16}N_2O_3S$	$C_{18}H_{15}ClN_2O_3S$	$C_{33}H_{26}N_2O_6S_2$	$C_{24}H_{21}NO_4S$
**MW**	283.34	377.50	265.29	326.37	340.40	374.84	610.70	419.49
**#Heavy atoms**	20	26	18	23	24	25	43	30
**#Aromatic heavy atoms**	12	6	6	12	12	12	24	18
**Fraction Csp3**	0.00	0.45	0.08	0.00	0.06	0.06	0.06	0.08
**#Rotatable bonds**	5	14	6	7	8	8	14	8
**#H-bond acceptors**	2	4	4	3	3	3	6	4
**#H-bond donors**	1	1	1	2	2	2	2	1
**MR**	83.02	107.09	68.64	91.57	94.84	99.85	170.46	121.66
**TPSA**	84.41	110.71	110.71	113.51	113.51	113.51	195.12	102.87

**iLOGP**	2.65	3.11	2.26	2.38	2.58	2.97	3.75	3.95
**XLOGP3**	3.23	4.96	1.24	2.23	2.16	2.79	5.93	5.02
**WLOGP**	2.83	4.08	0.96	2.23	1.95	2.60	5.08	4.51
**MLOGP**	3.15	3.38	1.40	2.32	2.29	2.79	4.18	3.60
**Silicos-IT Log P**	4.11	5.67	2.40	3.27	3.67	4.32	7.88	5.90
**Consensus Log P**	3.19	4.24	1.65	2.49	2.53	3.09	5.37	4.60
**ESOL Log S**	-3.75	-4.55	-2.12	-3.19	-3.15	-3.75	-6.85	-5.52
**ESOL Solubility (mg/ml)**	5.09e-02	1.06e-02	2.03e+00	2.10e-01	2.39e-01	6.68e-02	8.60e-05	1.27e-03
**ESOL Solubility (mol/l)**	1.80e-04	2.81e-05	7.64e-03	6.42e-04	7.03e-04	1.78e-04	1.41e-07	3.02e-06
**ESOL Class**	Soluble	Moderately soluble	Soluble	Soluble	Soluble	Soluble	Poorly soluble	Moderately soluble
**Ali Log S**	-4.68	-7.02	-3.16	-4.25	-4.18	-4.83	-9.80	-6.92
**Ali Solubility (mg/ml)**	5.98e-03	3.58e-05	1.82e-01	1.84e-02	2.27e-02	5.54e-03	9.63e-08	5.04e-05
**Ali Solubility (mol/l)**	2.11e-05	9.48e-08	6.87e-04	5.64e-05	6.66e-05	1.48e-05	1.58e-10	1.20e-07
**Ali Class**	Moderately soluble	Poorly soluble	Soluble	Moderately soluble	Moderately soluble	Moderately soluble	Poorly soluble	Poorly soluble
**Silicos-IT LogSw**	-4.48	-5.58	-2.05	-4.46	-4.86	-5.45	-9.19	-7.16
**Silicos-IT Solubility (mg/ml)**	9.34e-03	9.91e-04	2.38e+00	1.13e-02	4.71e-03	1.33e-03	3.99e-07	2.88e-05

**Silicos-IT Solubility (mol/l)**	3.30e-05	2.62e-06	8.95e-03	3.45e-05	1.38e-05	3.55e-06	6.53e-10	6.85e-08
**Silicos-IT class**	Moderately soluble	Moderately soluble	Soluble	Moderately soluble	Moderately soluble	Moderately soluble	Poorly soluble	Poorly soluble
**GI absorption**	High	High	High	High	High	High	Low	High
**BBB permeant**	No	No	No	No	No	No	No	No
**Pgp substrate**	No	No	No	No	No	No	No	No
**CYP1A2 inhibitor**	Yes	Yes	No	No	No	Yes	No	Yes
**CYP2C19 inhibitor**	Yes	Yes	No	No	Yes	Yes	No	Yes
**CYP2C9 inhibitor**	Yes	Yes	No	Yes	Yes	Yes	No	Yes
**CYP2D6 inhibitor**	No	No	No	No	No	No	No	No
**CYP3A4 inhibitor**	No	Yes	No	No	No	Yes	No	Yes
**log Kp (cm/s)**	-5.74	-5.08	-7.04	-6.71	-6.84	-6.61	-5.81	-5.29
**Lipinski #violations**	0	0	0	0	0	0	2	0
**Ghose #violations**	0	0	0	0	0	0	2	0
**Veber #violations**	0	1	0	0	0	0	2	0
**Egan #violations**	0	0	0	0	0	0	1	0
**Muegge #violations**	0	0	0	0	0	0	3	1
**Bioavailability Score**	0.55	0.55	0.55	0.55	0.55	0.55	0.17	0.55

**PAINS #alerts**	0	0	0	0	0	0	0	0
**Brenk #alerts**	3	3	3	3	3	3	3	3
**Leadlikeness #violations**	0	3	0	0	1	2	3	3
**Synthetic Accessibility**	2.38	3.24	2.10	2.36	2.49	2.53	4.00	3.08

References

(1) Pittala, V.; Vanella, L.; Salerno, L.; Di Giacomo, C.; Acquaviva, R.; Raffaele, M.; Romeo, G.; Modica, M. N.; Prezzavento, O.; Sorrenti, V. Novel Caffeic Acid Phenethyl Ester (Cape) Analogues as Inducers of Heme Oxygenase-1. *Curr Pharm Des* **2017**, *23* (18), 2657-2664.

(2) Li, M.; Li, J.; Zhang, T.; Zhao, Q.; Cheng, J.; Liu, B.; Wang, Z.; Zhao, L.; Wang, C. Syntheses, toxicities and anti-inflammation of H(2)S-donors based on non-steroidal anti-inflammatory drugs. *Eur J Med Chem* **2017**, *138*, 51-65.

(3) Mills, E. L.; Ryan, D. G.; Prag, H. A.; Dikovskaya, D.; Menon, D.; Zaslona, Z.; Jedrychowski, M. P.; Costa, A. S. H.; Higgins, M.; Hams, E.; et al. Itaconate is an anti-inflammatory metabolite that activates Nrf2 via alkylation of KEAP1. *Nature* **2018**, *556* (7699), 113-117.

(4) Kilkenny, C.; Browne, W. J.; Cuthill, I. C.; Emerson, M.; Altman, D. G. Improving bioscience research reporting: the ARRIVE guidelines for reporting animal research. *PLoS Biol* **2010**, *8* (6), e1000412.
